# Mucin architecture behind the immune response: design, evaluation and conformational analysis of an antitumor vaccine derived from an unnatural MUC1 fragment†
†Electronic supplementary information (ESI) available: Characterization of the new compounds, biological assays and MD simulations. See DOI: 10.1039/c5sc04039f


**DOI:** 10.1039/c5sc04039f

**Published:** 2015-12-15

**Authors:** Nuria Martínez-Sáez, Nitin T. Supekar, Margreet A. Wolfert, Iris A. Bermejo, Ramón Hurtado-Guerrero, Juan L. Asensio, Jesús Jiménez-Barbero, Jesús H. Busto, Alberto Avenoza, Geert-Jan Boons, Jesús M. Peregrina, Francisco Corzana

**Affiliations:** a Departamento de Química , Universidad de La Rioja , Centro de Investigación en Síntesis Química , Madre de Dios 53 , 26006 Logroño , Spain . Email: francisco.corzana@unirioja.es ; Email: jesusmanuel.peregrina@unirioja.es; b Complex Carbohydrate Research Center , University of Georgia , 315 Riverbend Road , Athens , Georgia 30602 , USA . Email: gjboons@ccrc.uga.edu; c BIFI , University of Zaragoza , BIFI-IQFR (CSIC) Joint Unit , Mariano Esquillor s/n , Campus Rio Ebro , Edificio I+D , Zaragoza , Spain; d Fundación ARAID , 50018 , Zaragoza , Spain; e Instituto de Química Orgánica General , IQOG-CSIC , Juan de la Cierva 3 , 28006 Madrid , Spain; f Structural Biology Unit , CIC bioGUNE , Parque Tecnológico de Bizkaia Building 801A , 48160 Derio , Spain; g IKERBASQUE , Basque Foundation for Science , 48011 Bilbao , Spain; h Department of Chemical and Physical Biology , Centro de Investigaciones Biológicas , CSIC , Ramiro de Maeztu 9 , 28040 Madrid , Spain

## Abstract

A tripartite cancer vaccine candidate, containing a quaternary amino acid (α-methylserine) in the most immunogenic domain of MUC1, has been synthesized and examined for antigenic properties in transgenic mice. The vaccine which is glycosylated with GalNAc at the unnatural amino acid, was capable of eliciting potent antibody responses recognizing both glycosylated and unglycosylated tumour-associated MUC1 peptides and native MUC1 antigen present on cancer cells. The peptide backbone of the novel vaccine presents the bioactive conformation in solution and is more resistant to enzymatic degradation than the natural counter part. In spite of these features, the immune response elicited by the unnatural vaccine was not improved compared to a vaccine candidate containing natural threonine. These observations were rationalized by conformational studies, indicating that the presentation and dynamics of the sugar moiety displayed by the MUC1 derivative play a critical role in immune recognition. It is clear that engineered MUC1-based vaccines bearing unnatural amino acids have to be able to emulate the conformational properties of the glycosidic linkage between the GalNAc and the threonine residues. The results described here will be helpful to the rational design of efficacious cancer vaccines.

## Abstract 2

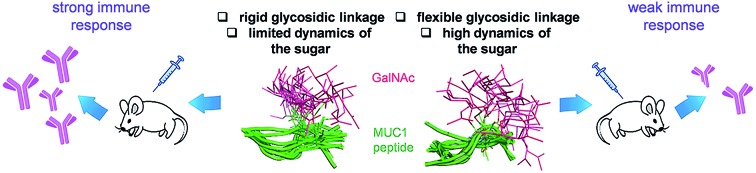
Presentation and dynamics of the sugar moiety in MUC1-based vaccines could play a crucial role in the elicitation of a strong immune response.

## Introduction

Mucin MUC1 is an *O*-glycoprotein strongly over-expressed in various tumours.^1^–^5^ In healthy tissues, the MUC1 backbone presents complex oligosaccharides, while in cancer cells it shows simple and truncated carbohydrates because of the incomplete glycosylation. As a result, different tumour-associated carbohydrate antigens (TACAs), such as the Tn determinant (α-*O*-GalNAc-Ser/Thr),^6^ are exposed to the immune system and can be recognized by different antibodies.^7^ Due to this unique characteristic, MUC1 derivatives are attracting great interest as a potential tool in developing therapeutic vaccines for the treatment of cancer.^3^,^8^–^10^ However, to date, none of them have succeeded in clinical trials^11^ due to the fact that most natural TACAs are tolerated by the immune system and glycosidases can reduce their *in vivo* bioavailability.^12^ One way to overcome this issue is through the use of chemical modifications of the antigens to generate non-natural determinants.^13^ A number of TACA mimics, comprising C-glycosides^14^–^16^ and S-glycosides,^17^–^19^ have been incorporated into carbohydrate-based vaccines. Additionally, the use of homoserine and β^3^-homothreonine conjugates^20^,^21^ to construct mucin-like glycopeptides and derivatives that incorporate fluorine atoms^22^,^23^ have been proposed.

Nevertheless, it is important to mention that the structural basis for the design of these vaccines remain unclear. From the view of molecular recognition, the Pro-Asp-Thr-Arg (PDTR) sequence comprises the minimal epitope for most of anti-MUC1 antibodies,^7^ such as SM3,^24^ which has a potential use in the early diagnosis and treatment of breast cancer.^25^ Although the crystal structure of this antibody in complex with a small peptide was reported some years ago,^25^ we have recently reported the X-ray structures of short glycopeptides bound to SM3.^26^ The analysis of these structures reveals that the threonine (Thr) residue of this epitope adopts a helix-like conformation and that its β-methyl is engaged in a hydrophobic contact with the surface of the antibody.

With these considerations in mind, we designed a novel Tn antigen mimic based on the quaternary amino acid α-methylserine (MeSer).^27^ This amino acid favors helix-like structures^28^ and has a methyl group at Cα that can establish the hydrophobic contact commented above with the antibody.

**Fig. 1:**
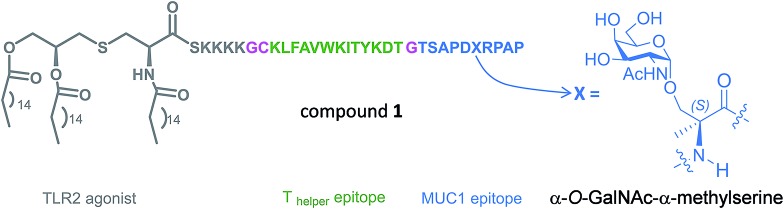
Novel vaccine incorporating the unnatural amino acid α-methylserine at the MUC1 epitope.

In the present work, we have incorporated this unnatural Tn into the most immunogenic domain of a MUC1 fragment, designing a three-component cancer vaccine (Fig. 1), similar to that previously reported by Boons and co-workers.^8^ We have demonstrated that the unnatural glycosylated epitope exhibits better stability in human serum when compared to the natural derivative. The novel vaccine is able to elicit a potent immune response in transgenic mice, recognizing both glycosylated and unglycosylated tumour-associated MUC1 derivatives and native MUC1 antigen present on cancer cells. The effectiveness of this vaccine is comparable (but not better) to that observed for its homologue derivative with threonine. To explain the experimental data, we have performed an extensive conformational analysis on the vaccine in the free state in water as well as bound to phospholipid-based liposomes. The analysis involves the use of NMR and Molecular Dynamics (MD) simulations. The conformational studies point out that the extra flexibility of the side chain and the glycosidic linkage of the unnatural GalNAc-MeSer fragment have an unfavorable impact on the molecular recognition by the immune system. This information reinforces the idea that the design of more efficient vaccines based on MUC1 has to involve the use of glycopeptides that can imitate the conformational preferences of the aberrantly glycosylated natural MUC1 epitope.

## Results and discussion

**Scheme 1:**
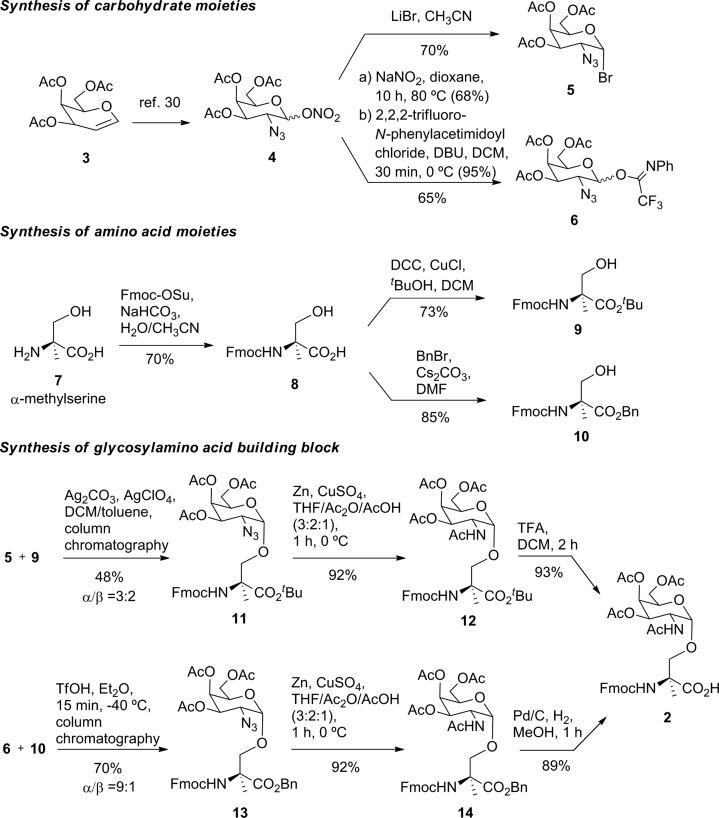
Synthesis of building block **2**.

Prior to the preparation of vaccine candidate **1**, we optimized the synthesis of the unnatural building block **2** (Scheme 1) in a large scale. To this end, we developed two divergent synthetic routes that are summarized in Scheme 1. The first one uses derivative **5** as a glycosyl donor, which was easily prepared from compound **4** and following the methodology described in the literature.^29^ Derivative **4** was obtained from commercially available 3,4,6-tri-*O*-acetyl-d-galactal **3** (see ref. 30). The adequately protected α-methylserine **9**, as *N*-Fmoc^31^ and *tert*-butyl ester^32^ protecting groups, was synthesized starting from α-methylserine **7**, obtained by methodology described by us.^33^ Initially, to carry out the glycosylation of protected amino acid **9** with derivative **5**, we examined the method of Liebe and Kunz^34^ using AgCO_3_ and AgClO_4_ in a mixture of toluene and dichloromethane (DCM), obtaining a mixture of α/β anomers in a ratio of 3 : 2 in a moderate yield. The α-anomer **11** was purified by column chromatography and its azide group was transformed into an acetamido group by reduction with Zn in the presence of CuSO_4_ to give compound **12** in an excellent yield. The *tert*-butyl ester group of this compound was removed to afford the required building block **2**. In order to improve the yield of the glycosylation reaction, as well as the anomeric ratio, we used a modification of the trichloroacetimidate method.^35^ Therefore, the imidate-derivative **6** was prepared by hydrolysis of the nitro group of compound **4** (ref. 36), followed by treatment with 2,2,2-trifluoro-*N*-phenylacetimidoyl chloride in the presence of DBU. The use of the acid glycosylation method forced us to prepare a different and convenient protected quaternary amino acid. *N*-Fmoc-α-methylserine **8** was converted into benzyl ester derivative **10** using benzyl bromide in the presence of cesium carbonate. The glycosylation of this compound with imidate **6** was carried out with triflic acid in diethyl ether at –40 °C for 15 min, giving a 70% yield of α/β anomers in a ratio 9 : 1. α-Anomer **13** was purified by silica gel column chromatography and its azide group was reduced to acetamido group to give compound **14**.

The benzyl ester of the latter compound was removed by hydrogenolysis, giving building block **2** in a good overall yield (Scheme 1).

**Scheme 2:**
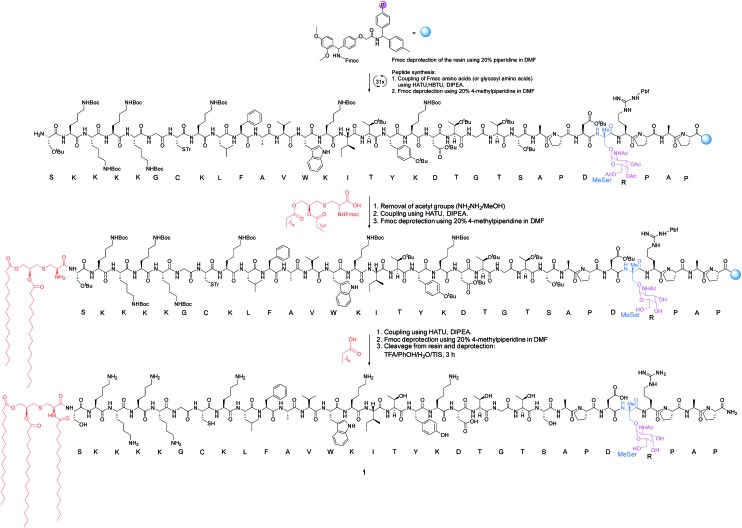
Synthesis of vaccine **1**. The incorporation of building block **2** and the assembly of Fmoc-Pam_2_-Cys-OH were manually performed. HBTU = *N*,*N*,*N*′,*N*′-tetramethyl-*O*-(1*H*-benzotriazol-1-yl)uronium hexafluorophosphate. HATU = 1-[bis(dimethylamino)methylene]-1*H*-1,2,3-triazolo[4,5-*b*]pyridinium 3-oxid hexafluorophosphate. DIPEA = *N*,*N*-diisopropylethylamine. TFA = trifluoroacetic acid. TIS = triisopropylsilane. DMF = *N*,*N*-dimethylformamide.

The synthesis of vaccine candidate **1** was carried out by microwave-assisted solid phase peptide synthesis (MW-SPPS) employing a Rink amide AM LL resin, Fmoc-protected amino acids and derivative **2** (Scheme 2).^35^ The incorporation of building block **2** and the assembly of Fmoc-Pam_2_-Cys-OH were manually performed under microwave-assisted coupling conditions.

We examined then the stability of the unnatural MUC1 derivative in human plasma. To this end, we synthesized glycopeptides APDThr(α-d-GalNAc)RP and APDMeSer(α-d-GalNAc)RP (ESI†). The degradation of the glycopeptide containing threonine was faster (around 1.8-fold) compared to the unnatural compound. We hypothesized that this resistance may translate to an increase in *in vivo* stability and bioavailability and hence lead to stronger and longer-lasting antigenic responses.

To examine the immunity of lipoglycopeptide **1**, it was incorporated into phospholipid-based small vesicles (ESI†). The aliphatic chain of the palmitic acid in the TLR2 agonist favours the incorporation of the vaccine into liposomes, which may enhance the circulation time of the vaccine and allows for the presentation of the glycopeptide epitope in a multivalent manner.^8^

**Fig. 2:**
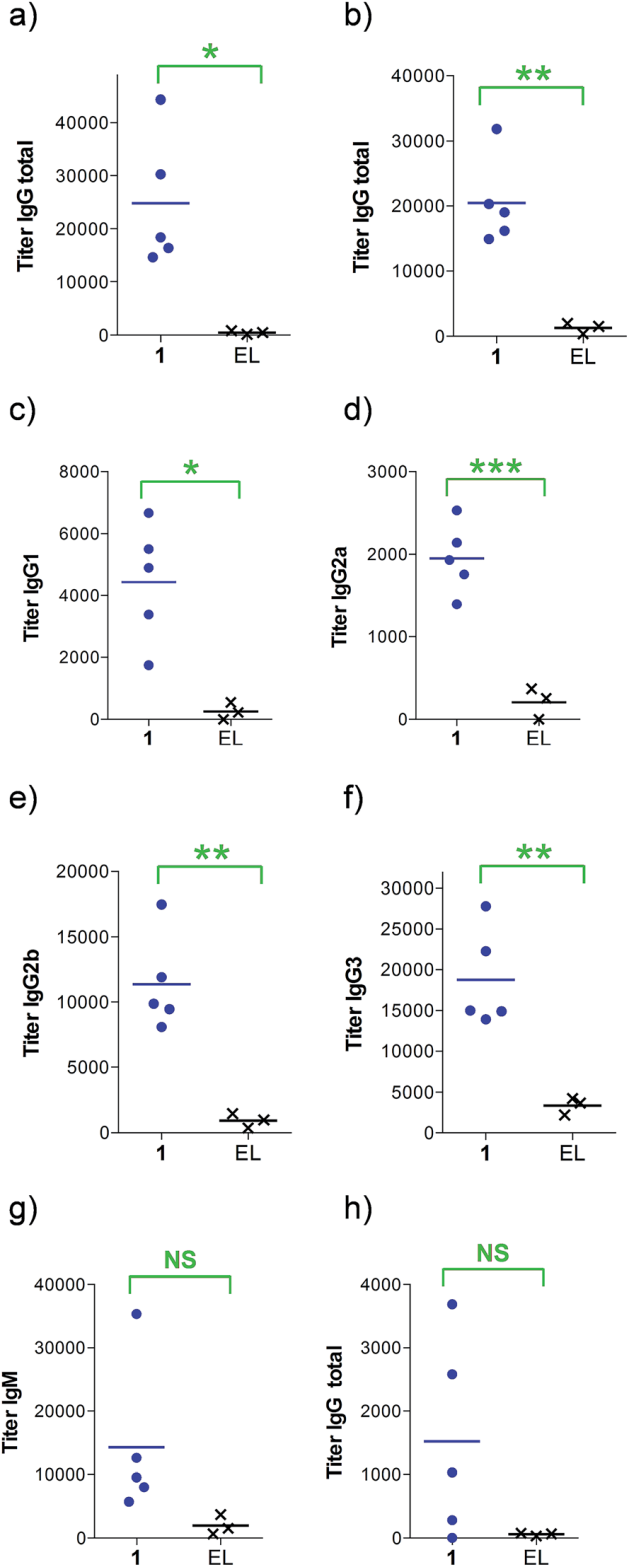
Anti-MUC1 and anti-T_helper_ antibody titers after (a) 3 or (b–h) 4 immunizations with either empty liposomes (*n* = 3) or vaccine **1** in liposomes (*n* = 5) as indicated. ELISA plates were coated with (a–g) BSA-MI-CTSAPDT(α-d-GalNAc)RPAP conjugate or (h) NeutrAvidin-biotin-T_helper_ and titers were determined by linear regression analysis, plotting dilution *vs.* absorbance. Titers were defined as the highest dilution yielding an optical density of 0.1 or greater over that of normal control mouse sera. EL stands for empty liposomes. MI stands for maleimide. Each data point represents the titer for an individual mouse and the horizontal lines indicate the mean for the group of mice. Asterisks indicate statistically significant difference (*** *P* < 0.001, ** *P* < 0.01, * *P* < 0.05) and NS indicates no significant difference.

Next, groups of MUC1.Tg mice (C57BL/6; H-2b) that express human MUC1 (ref. 37) were immunized four times at biweekly intervals with liposomal preparations of compound **1** and empty liposomes as control. One week after the last immunization, the mice were sacrificed and the serum harvested. Mice immunized with **1** elicited a specific anti-MUC1 IgG antibody response (Table 1 and Fig. 2), which was significantly different from the control group immunized with empty liposomes (EL). Sub-typing of the IgG antibodies shows significant IgG1 and IgG2a,b responses, indicating a mixed Th1/Th2 response.

**Table 1:** ELISA anti-MUC1 and anti-T_helper_ antibody titers^*a*^ after 3rd & 4th immunization

Immunization^*b*^	1	EL^*d*^
IgG total, MUC1, 3rd imm	18 400	800
IgG total, MUC1, EP^*c*^	19 000	1500
IgG1, MUC1, EP	4900	600
IgG2a, MUC1, EP	1900	0
IgG2b, MUC1, EP	9900	1000
IgG3, MUC1, EP	15 000	400
IgM, MUC1, EP	9600	100
IgG total, T_helper_, EP	1000	100

^*a*^Anti-MUC1 and anti-T_helper_ antibody titers are presented as median values for groups of mice. ELISA plates were coated with BSA-MI-CTSAPDT(α-d-GalNAc)RPAP conjugate for anti-MUC1 antibody titers or NeutrAvidin-biotin-T_helper_ for anti-T_helper_ antibody titers. Titers were determined by linear regression analysis, with plotting of dilution *versus* absorbance. Titers are defined as the highest dilution yielding an optical density of 0.1 or greater relative to normal control mouse sera.

^*b*^Liposomal preparations were employed.

^*c*^EP stands for endpoint (serum samples after 4 immunizations).

^*d*^EL stands for empty liposomes. MI stands for maleimide. Individual anti-MUC1 titers for IgG total, IgG1, IgG2a, IgG2b, IgG3 and IgM, and anti-T_helper_ epitope for IgG total are reported in Fig. 2.

The observed high IgG3 titer is typical of an anti-carbohydrate response. IgM antibody titers were low and not significantly different from the control. Low amounts of IgG antibodies raised against the T_helper_ epitope were measured; indicating that candidate vaccine **1** does not suffer from significant immune suppression.

**Fig. 3:**
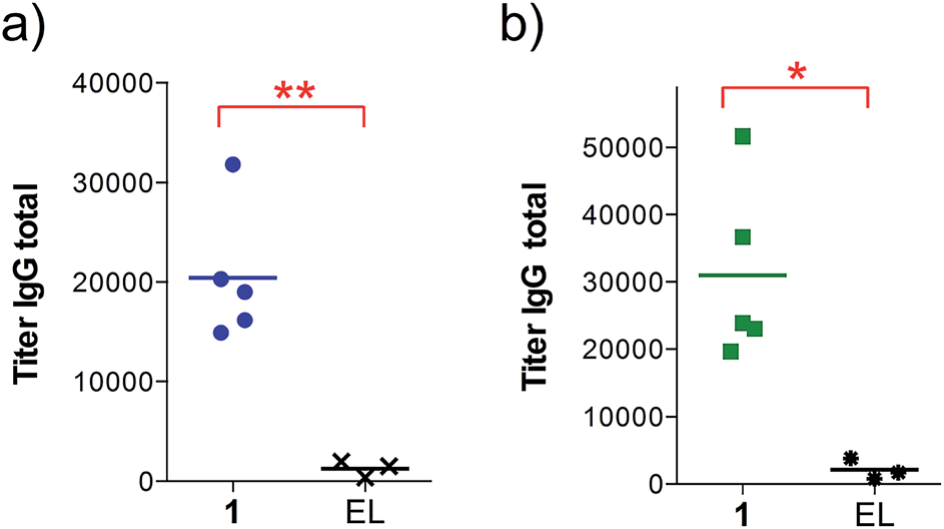
Anti-MUC1 (glycosylated and unglycosylated) antibody titers after 4 immunizations with either empty liposomes (*n* = 3) or vaccine **1** in liposomes (*n* = 5) as indicated. ELISA plates were coated with (a) BSA-MI-CTSAPDT(α-d-GalNAc)RPAP conjugate for anti-MUC1(Tn) titers or (b) BSA-MI-CTSAPDTRPAP conjugate for anti-unglycosylated MUC1 titers. EL stands for empty liposomes. MI stands for maleimide. Each data point represents the titer for an individual mouse and the horizontal lines indicate the mean for the group of mice. Asterisks indicate statistically significant difference (** *P* < 0.01, * *P* < 0.05).

We found that the antibodies produced by vaccine **1** could recognize glycosylated and unglycosylated MUC1 epitopes with similar titers (Fig. 3), indicating that antibodies recognize the peptide and not the glycan.

**Fig. 4:**
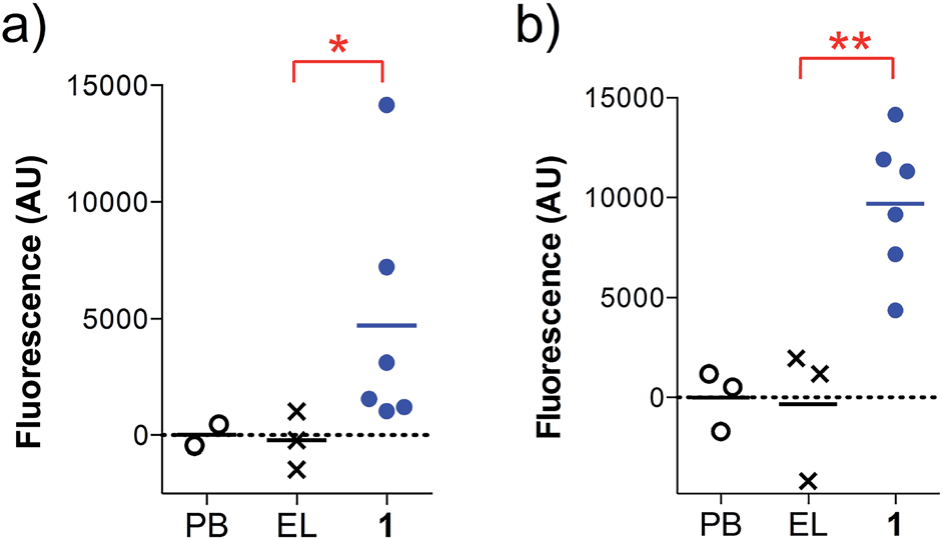
Cell recognition analysis for specific anti-MUC1 antibodies. Serum samples (1 : 50 diluted) were incubated with (a) MCF7 or (b) C57mg.MUC1 cells. After incubation with FITC-labeled anti-mouse IgG antibody, the fluorescence intensity was assessed in cell lysates. AU indicates arbitrary fluorescence units; PB stands for pre-bleed; EL stands empty liposomes. Each data point represents an individual mouse and the horizontal lines indicate the mean for the group of mice. Asterisks indicate statistically significant difference (** *P* < 0.01, * *P* < 0.05).

In addition, the ability of the mouse antisera to recognize the MUC1 antigen expressed on cancer cells was investigated. Serum samples were added to MUC1 expressing MCF7 human breast cancer and C57mg.MUC1 mammary gland tumor cells, and recognition was established using a FITC-labeled anti-mouse IgG antibody. As shown in Fig. 4, antisera obtained from immunizations with vaccine **1** displayed recognition of MUC1 tumour cells, whereas no binding was observed for the controls.

Of note, vaccine **1** did not improve the effectiveness of a previously reported natural threonine-containing derivative^8^ (ESI†). Prompted by these results, we investigated whether the unnatural scaffold influences the presentation of the epitope. To this end, we ran 100 ns MD simulations on phospholipid-based liposomes loaded with only a lipoglycopeptide **1** molecule to simplify the system. For comparative purposes, we performed also the MD simulation on the threonine analogue. According to these simulations, which start with an extended conformation of the peptidic backbone, both lipoglycopeptides have a clear tendency to adopt folded conformations, allowing the sugar moiety to be close to the liposome surface at the end of the MD simulations. This feature is more marked in the analogue with threonine.

**Fig. 5:**
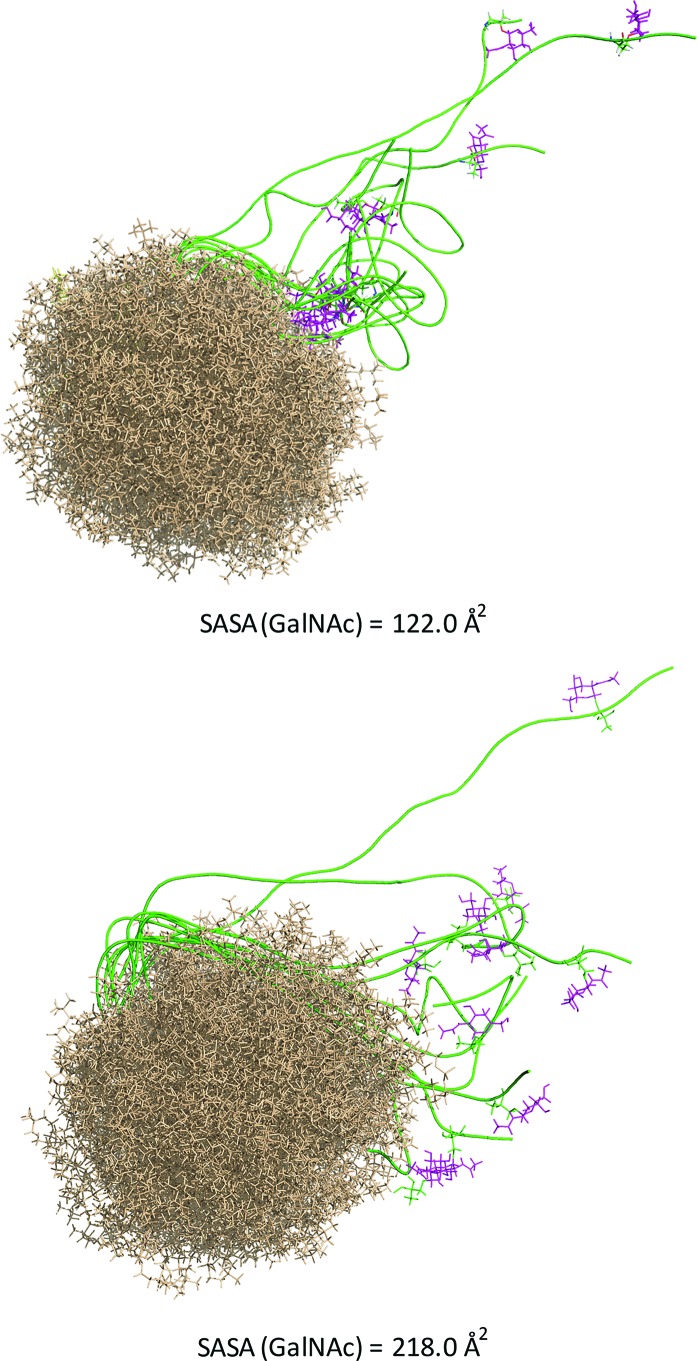
Ensembles obtained from 100 ns MD simulations on liposomes consisting of 65 dodecylphosphocholine lipids loaded with the threonine-containing lipoglycopeptide (upper panel) or derivative **1** (bottom panel). The peptide backbone is shown in green, the GalNAc unit in purple and the liposomes in brown. The solvent-accessible surface area (SASA) calculated for the GalNAc residues is shown.

The solvent-accessible surface area (SASA) calculated for the GalNAc residue, which gives an idea of its accessibility, is *ca.* 1.8-fold higher for the unnatural vaccine (Fig. 5), indicating that the unnatural scaffold does not hinder the Tn-mimicry to interact with the immune system.

**Fig. 6:**
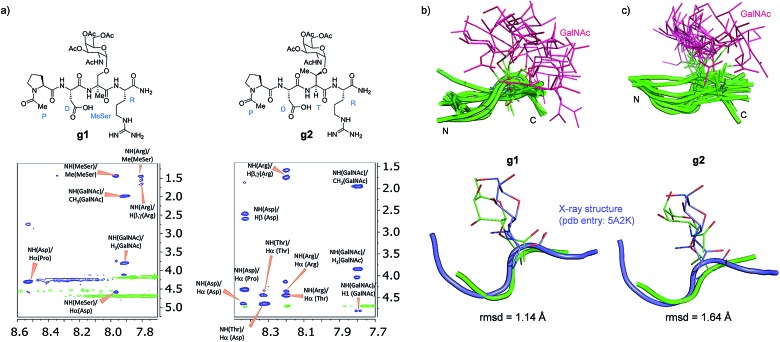
(a) Glycopeptides **g1** and **g2** and sections of the 500 ms 2D NOESY spectrum (400 MHz) in H_2_O/D_2_O (9 : 1) at 25 °C and pH = 5.6 showing the amide cross-peaks. Diagonal peaks and exchange cross-peaks connecting NH protons and water are negative (green color). The NOE contacts are represented as positive cross-peaks (blue color). Ensembles obtained for glycopeptides **g1** (b) and **g2** (c) through the 20 ns MD-tar simulations in water, together with the main conformation found in solution for the backbone of the glycopeptides (lower panels). The backbone of **g1** and **g2** are superimposed onto the geometry of the APDThr(α-*O*-GalNAc)RP glycopeptide in complex with SM3 antibody (PDB entry: ; 5A2K). rmsd = root-mean-square deviation.

Attending to our simulations, this result does not explain the experimental evidence. Accordingly, we focused our conformational analysis on the epitope fragments. Thus, glycopeptides **g1** and **g2** (Fig. 6a) were synthesized and subjected to detailed conformational analysis by combining NMR and MD simulations. The proton–proton distances obtained from 2D-NOESY experiments (Fig. 6a) were used as restraints in MD simulations (MD-tar).^38^,^39^ This type of calculation provides a distribution of low energy conformers able to reproduce the experimental data. The good agreement obtained in our simulations between the experimental and calculated distances validates the theoretical study (see ESI†).

**Fig. 7:**
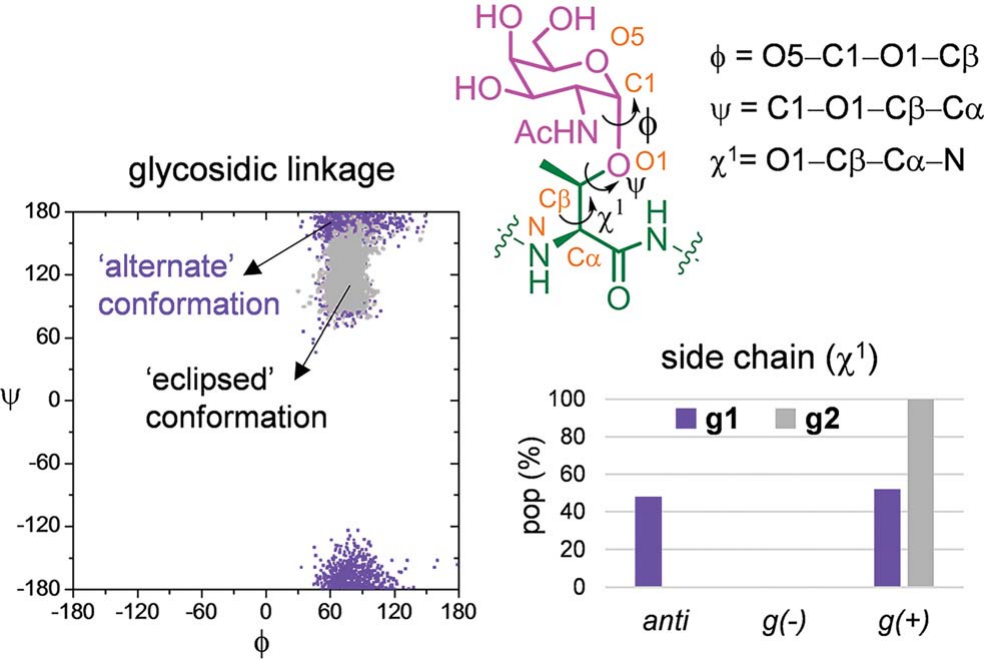
Differences obtained from the MD-tar simulations between the glycosidic linkage and the side chain of α-methylserine and threonine residues in compounds **g1** and **g2**, respectively.

Fig. 6b and c show the ensemble obtained from 20 ns MD-tar simulations in explicit water. The unnatural residue in compound **g1** adopts a helix-like conformation in water, which is validated by the NOE cross-peaks between the methyl group of MeSer and NH protons of MeSer and Arg residues (Fig. 6a). The folded conformation adopted by the unnatural residue is in agreement with previous studies on other glycopeptides carrying this quaternary amino acid.^27^ However, the presence of α-methyl group provides flexibility to the peptide backbone. The most populated conformer (30%) is similar to the bioactive conformation found in the crystal structures.^26^ This conformation is barely populated in the free state for glycopeptide **g2**, which exhibits an extended conformation (around 78%) for the backbone, in agreement with previous structural studies on MUC1-derived glycopeptides modified by GalNAc.^26^,^40^–^43^ Concerning the glycosidic linkage, it adopts the ‘alternate’ conformation in the unnatural glycopeptide **g1**, with *φ* torsional angle around 80°, in agreement with the exo-anomeric effect, and with the *ψ* dihedral close to 180° (Fig. 7). This geometry of the glycosidic linkage is, as expected,^44^ observed in the analogue glycopeptide with serine also studied in this work (ESI†). In contrast, the ‘eclipsed’ conformation, with *φ* around 120°, is preferred in glycopeptide **g2** (ref. 45). This particular conformation is characterized by a NOE cross-peak between the amide groups of GalNAc and Thr (ESI†). Consequently, the carbohydrate moiety adopts different orientations with respect to the peptide backbone in the investigated compounds. Regarding the side chain of the glycosylated residue, while in **g2** it is locked, showing a value close to 60°, in glycopeptide **g1** the *χ*^1^ torsional angle is rather flexible, showing two similar populated conformers, with values around 60° [*g*(+)] and 180° [*anti*] (Fig. 7). These conformational differences were also observed when peptide fragments, **g1** (amino acids in blue in Fig. 1) and **g2** (natural epitope), are part of the 10-mer MUC1 derivatives, indicating the generality of the different conformational behavior. These findings were deduced from the 100 ns MD simulations carried out on the liposomes (Fig. 5 and ESI†). Therefore, although the unnatural glycopeptide exhibits a higher population of the bioactive backbone conformation with respect to the natural epitope, it displays a higher flexibility at the sugar moiety. Presumably, the extra flexibility of the unnatural epitope will have a negative impact on the molecular recognition by the immune system. This hypothesis might be validated by the excellent results recently reported by Nativi and co-workers with a non-natural vaccine based on a conformational restricted Tn antigen.^13^ In summary, our data reinforces the idea that the design of more efficient cancer vaccines has to involve the use of glycopeptides that can emulate the peculiar conformational preferences of the α-GalNAc-Thr glycosidic linkage,^45^ found in aberrantly glycosylated natural MUC1 epitope.

## Conclusions

A MUC1-based vaccine containing the quaternary amino acid α-methylserine at the most immunogenic domain has been synthesized and the immunogenicity examined in transgenic mice. The vaccine elicits robust antibody titers recognizing glycosylated and unglycosylated tumour-associated MUC1 derivatives and native MUC1 antigen present on cancer cells. In spite of the peptide backbone of the novel vaccine presenting the bioactive conformation in solution and it being more resistant to enzymatic degradation than the natural one, the immune response elicited by this unnatural vaccine is not superior to the threonine analogue. To rationalize these observations, we have performed conformational studies on the unnatural vaccine and on its natural counterpart. These studies indicate that the presentation and dynamics of the sugar moiety displayed by the MUC1 derivative may be critical for a strong immune response. The present work offers a new way to engineer MUC1-based vaccines based on the conformational behavior of their components. The designed unnatural amino acids have to be able to emulate the particularities of the glycosidic linkage between the GalNAc and the threonine residues. Our results provide foundational information for the rational design of carbohydrate-based vaccines against cancer.

## Supplementary Material

Supplementary information
