# Supplementary material for: Mucin architecture behind the immune response: design, evaluation and conformational analysis of an antitumor vaccine derived from an unnatural MUC1 fragment
Source: Chem Sci. 2015 Dec 15;7(3):2294–301. doi: 10.1039/c5sc04039f (PMC5977504; doi:10.1039/c5sc04039f)
Supplement: Supplementary file 1 [file SC-007-C5SC04039F-s001.pdf]

Electronic Supplementary Material (ESI) for Chemical Science.
This journal is © The Royal Society of Chemistry 2015

Supporting Information

for

Mucin Architecture behind the Immune Response: Design, Evaluation and Conformational Analysis of an Antitumor Vaccine Derived from an Unnatural MUC1 Fragment

Nuria Martínez-Sáez, Nitin T. Supekar, Margreet A. Wolfert, Iris A. Bermejo, Ramón Hurtado-Guerrero, Juan L. Asensio, Jesús Jiménez-Barbero, Alberto Avenoza, Jesús H. Busto, Geert-Jan Boons, Jesús M. Peregrina, and Francisco Corzana

Experimental

Reagents and general procedures. Commercial reagents were used without further purification. Solvents were dried and redistilled prior to use in the usual way. All reactions were performed in oven-dried glassware with magnetic stirring under an inert atmosphere unless noted otherwise. High-resolution electrospray mass (ESI) spectra were recorded on a microTOF spectrometer; accurate mass measurements were achieved by using sodium formate as an external reference.

NMR experiments. ^1H and ^{13}C NMR spectra were measured with a 400 MHz spectrometer at 298K with TMS as the internal standard. Multiplicities are quoted as singlet (s), broad singlet (br s), doublet (d), doublet of doublets (dd), triplet (t), or multiplet (m). Signals of spectra were assigned using COSY and HSQC. All NMR chemical shifts (δ) were recorded in ppm and coupling constants (*J*) were reported in Hz. The results of these experiments were processed with MestreNova software. Magnitude-mode ge-2D COSY spectra were acquired with gradients by using the cosygpqf pulse program with a pulse width of 90°. Phase-sensitive ge-2D HSQC spectra were acquired by using z-filter and selection before t1 removing the decoupling during acquisition by use of the invigpndph pulse program with CNST2 (JHC)=145.

2D NOESY experiments. NOESY experiments were recorded on a Bruker Avance 400 spectrometer at 298 K and pH 5.6 in H_2O/D_2O (9:1). The experiments were conducted by using phase-sensitive ge-2D NOESY with WATERGATE for H_2O/D_2O (9:1) spectra. NOEs intensities were normalized with respect to the diagonal peak at zero mixing time. Distances involving NH protons were semi-quantitatively determined by integrating the volume of the corresponding cross-peaks. The number of scans used was 16 and the mixing time was 500 ms.

Solid-phase peptide synthesis (SPPS). Glycopeptides were synthesized by stepwise solid-phase peptide synthesis using the Fmoc strategy on Rink Amide MBHA resin (0.1 mmol). The glycosylated amino acid building block **2** and the assembly of Fmoc-Pam$_2$-Cys-OH was coupled manually using 2 equiv. and activated with HBTU, while the other Fmoc amino acids were coupled in the automated mode in an Applied Biosystems 433A peptide synthesizer using 10 equiv. and HBTU. The *O*-acetyl groups of the GalNAc moiety were deprotected in a mixture of NH_2NH_2/MeOH (7:3). The glycopeptides were then released from the resin, and all acid sensitive side-chain protecting groups simultaneously removed using 95% TFA, 2% TIS, 1% EDT, 2% H_2O, followed by precipitation with ether. Finally, all the compounds were purified by HPLC on a Waters Delta Prep chromatograph [Phenomenex Luna C18(2) column (10 μ, 250 mm × 21.2 mm)].

Synthesis of compound 1. Synthesis of glycolipopeptide vaccine **1** was done by similar protocol as mentioned above with further modifications. Glycolipopeptide **1** was prepared employing compound **2** as a building block using MW-SPPS on Rink Amide AM LL Resin (0.1 mmol) using an automated CEM-Liberty instrument equipped with a UV-detector and a CEM-Discover SPS instrument. Side chain protection was as follows: Fmoc-L-Asp(O^{t}Bu)-OH, Fmoc-L-Cys(Trt)-OH, Fmoc-L-Lys(*N*-ε-*tert*-Boc)-OH, Fmoc-L-Ser(O^{t}Bu)-OH, Fmoc-L-Thr(O^{t}Bu)-OH, Fmoc-L-Tyr(O^{t}Bu)-OH. Glycosylated amino acid, compound **2** (87 mg, 0.13 mmol) was dissolved in DMF (2 mL) and HATU (51 mg, 0.13 mmol) and DIPEA (67 µL, 0.4 mmol) were premixed for 2 min and were added to the resin. The manual microwave-irradiated coupling reaction was monitored by Kaiser test and was complete after 10 min. The peptide was then elongated under MW-SPPS conditions described above until the final serine residue; the remaining steps performed manually. The resin was then treated with 60% hydrazine in methanol for 2 h. The resin was washed thoroughly with DMF (5 mL x 2), DCM (5 mL x 2) and MeOH (5 mL x 2) and then dried in vacuum. The resin was swollen in DCM (5 mL) for 1 h. *N*-α-Fmoc-*R*-(2,3-bis (palmitoyloxy)-(2*R*-propyl)-(*R*)-cysteine (180 mg, 0.2 mmol) was dissolved in DMF (5 mL). HATU (76 mg, 0.2 mmol) and DIPEA (67 µL, 0.4 mmol) were premixed for 2 min and added to the resin. The microwave-irradiated coupling reaction was monitored by the Kaiser test and was complete after 10 min. Upon completion of the coupling, the *N*-α-Fmoc group was cleaved using 20% 4-methyl piperidine in DMF (5 mL) under microwave irradiation. Palmitic acid (52 mg, 0.2 mmol) was coupled to the free amine as described above using HATU (76 mg, 0.2 mmol) and DIPEA (67 µL, 0.4 mmol) in DMF. The resin was washed thoroughly with DMF (5 mL x 2), DCM (5 mL x 2) and MeOH (5 mL x 2), and then dried in *vacuo*. The resin was swelled in DCM (5 mL) for 1 h, after which it was treated with reagent B (TFA 88%, water 5%, phenol 5% and TIS 2.5%; 10 mL) for 2 h. The resin was filtered and washed with neat TFA (2 mL). The filtrate was concentrated in *vacuo* to approximately 1/3 of its original volume. The peptide was precipitated using diethyl ether (0 °C, 30 mL) and recovered by centrifugation at 3,000 rpm for 15 min. The crude glycolipopeptide was purified by HPLC on a Jupiter analytical C-4 reversed phase column using a linear gradient of 0-100% solvent B (95% acetonitrile + water + 0.1% TFA) in A (95% water + acetonitrile + 0.1% TFA) over 40 min, and the appropriate fractions (t_R = 31.50 min) were lyophilized to afford **1**. MALDI-ToF/MS: m/z for $C_{217}H_{367}N_{45}NaO_{53}S_2$ $[M+Na]^+$ calc 4538.6745, found 4538.4663.

Synthesis of compound g1. Following SPPS methodology with Fmoc-L-Pro-OH (337 mg, 1 mmol), Fmoc-L-Asp(O^{t}Bu)-OH (412 mg, 1 mmol), compound **2** (201 mg, 0.30 mmol) and Fmoc-L-Arg(Pbf)-OH (649 mg, 1 mmol), glycopeptide **g1** was obtained with a 94% yield after purification by reversed-phase HPLC and lyophilization. t_R (Phenomenex Luna C18 (2), 21.20×250mm, Grad: acetonitrile/water+0.1% TFA (2:98) → (15:85), 30 min, λ = 212nm): 19.85 min. ^{1}H NMR (400 MHz, D_2O) δ = 1.45 (s, 3H, CH_3 MeSer), 1.50-1.62 (m, 2H, Hγ Arg), 1.65-1.76 (m, 1H, Hβ Arg), 1.75-1.95 (m, 4H, Hβ Pro , 2 Hγ Pro, Hβ Arg), 1.99 (s, 3H, $COCH_3$), 2.07 (s, 3H, $COCH_3$), 2.17-2.28 (m, 1H, Hβ Pro), 2.67-2.86 (m, 2H, Hβ Asp), 3.10-3.16 (m, 2H, Hδ Arg), 3.54-3.64 (m, 2H, Hδ Pro), 3.66-3.71 (m, 2H, H6), 3.74-3.94 (m, 4H, 2Hβ MeSer,

H5, H3), 3.89-3.92 (m, 1H, H4), 4.12 (dd, 1H, *J* = 11.0, 3.7 Hz, H2), 4.18 (dd, 1H, *J* = 9.5, 5.0 Hz, Hα Arg,), 4.32 (dd, 1H, *J* = 8.6, 4.7 Hz, Hα Pro), 4.55 (t, 1H, *J* = 6.6 Hz, Hα Asp), 4.80 (d, 1H, *J* = 3.7 Hz, H1). ^{1}H NMR [400 MHz, D_2O/H_2O (9:1)] δ = 7.80 (d, 1H, *J* = 7.6 Hz, NH Arg), 7.91 (d, 1H, *J* = 9.0 Hz, NH GalNAc), 7.96 (s, 1H, NH MeSer), 8.53 (d, 1H, *J* = 8.0 Hz, NH Asp). ^{13}C NMR (100 MHz, D_2O) δ 20.4 (CH_3 MeSer), 21.5 (CO$\underline{C}H_3$), 22.0 (CO$\underline{C}H_3$), 24.3, 24.6 (Cγ Pro, Cγ Arg), 27.7 (Cβ Arg), 29.9 (Cβ Pro), 35.6 (Cβ Asp), 40.4 (Cδ Arg), 48.7 (Cδ Pro), 49.7 (C2), 50.4 (Cα Asp), 53.3 (Cα Arg), 59.9 (Cα Pro), 60.3(Cα MeSer), 61.2 (C6), 67.6 (C3), 68.4 (C4), 69.3(Cβ Ser), 71.4 (C5), 97.4 (C1), 156.8 (C=N), 172.0, 173.4, 174.2, 174.5, 175.1, 176.3 (7 CO). HRMS (ESI+): calcd for $C_{29}H_{49}N_9O_{13}$ (MH+) 732.3523, found (MH^+) 732.3515.

Synthesis of compound g2. Following SPPS methodology with Fmoc-L-Pro-OH (337 mg, 1 mmol), Fmoc-L-Asp(O^{t}Bu)-OH (412 mg, 1 mmol), Fmoc-Thr[α-D-GalNAc(Ac)$_3$]-OH (201 mg, 0.30 mmol, ref. S1) and Fmoc-L-Arg(Pbf)-OH (649 mg, 1 mmol), glycopeptide **g2** was obtained with a 95% yield after purification by reversed-phase HPLC and lyophilization. t_R (Phenomenex Luna C18 (2), 21.20×250mm, Grad: acetonitrile/water+0.1% TFA (2:98) → (15:85), 30 min, λ = 212nm): 16.95 min. ^{1}H NMR (D_2O) *δ* (ppm): 1.19 (d, 1H, *J*=6.3 Hz, CH_3 Thr), 1.52 – 1.64 (m, 2H, Hγ Arg), 1.65 – 1.82(m, 2H, Hβ Arg), 1.83 – 1.95 (m, 3H, Hβ Pro , 2 Hγ Pro), 1.97 (s, 3H, $COCH_3$), 2.05 (s, 3H, $COCH_3$), 2.17 – 2.27 (m, 1H, Hβ Pro), 2.71 – 2.94 (m, 2H, Hβ Asp), 3.14 (t, 2H, *J*=6.8 Hz, Hδ Arg), 3.51 – 3.64 (m, 2H, Hδ Pro), 3.58 – 3.78 (m, 2H, H_{6S}), 3.84 (dd, 1H, *J*=11.0, 3.1 Hz, H_{3S}), 3.89 – 3.92 (m, 1H, H_{4S}), 3.92 – 3.98 (m, 1H, H_{5S}), 4.05 (dd, 1H, *J*=11.0, 3.8 Hz, H_{2S}), 4.18 – 4.23 (m, 1H, Hα Arg), 4.27 – 4.36 (m, 2H, Hβ Thr, Hα Pro), 4.46 (d, 1H, *J*=1.8 Hz, Hα Thr), 4.77 (t, 1H, *J*=6.9 Hz, Hα Asp), 4.81 (d, 1H, *J*=3.8 Hz, H_{1S}). ^{1}H NMR (H_2O/ D_2O) *δ* (ppm): amide region 6.49 – 6.73 (m, 2H, NH_{Gnd} Arg), 6.98 (s, 1H, NH_2), 7.27 – 7.39 (m, 2H, NH_{Gnd} Arg), 7.32 (s, 1H, NH_2), 7.79 (d, 1H, *J*= 9.6Hz, NH_S), 8.20 (d, 1H, *J*= 6.6 Hz, NH Arg), 8.32 (d, 1H, *J*= 8.5 Hz, NH Thr), 8.43 (d, 1H, *J*= 6.8 Hz, NH Asp). ^{13}C NMR (D_2O) *δ* (ppm): 18.2 (CH_3 Thr), 21.4 ($COCH_3$), 22.5 ($COCH_3$), 24.2, 24.4 (Cγ Pro, Cγ Arg), 28.3 (Cβ Arg), 29.9 (Cβ Pro), 35.5 (Cβ Asp), 40.4 (Cδ Arg), 48.6 (Cδ Pro), 49.7 (C_{2S}), 50.1 (Cα Asp), 53.2 (Cα Arg), 57.3 (Cα Thr), 60.1 (Cα Pro), 61.2 (C_{6S}), 68.0 (C_{3S}), 68.5 (C_{4S}), 71.4 (C_{5S}), 75.9 (Cβ Thr), 98.7 (C_{1S}), 156.8 (CN), 171.2, 173.0, 173.2, 174.2, 174.4, 174.7, 175.9 (7 CO). HRMS (ESI+) m/z: calcd. for $[M+H]^+$: 732.3523 found: 732.3509

Synthesis of compound 5. This compound was obtained following the methodology reported in the literature starting from the commercially available derivative **3** (ref. S2).

Synthesis of compound 6. To a solution of compound **4**, previously obtained from compound **3**, (1.50 g, 3.99 mmol) in dioxane (15 mL), $NaNO_2$ (1.93 g, 27.9 mmol) dissolved in water (4 mL) was added. After stirring the reaction mixture at 80°C for 10 h, cold water (10 mL) was added and the mixture was extracted with dichloromethane (25 mL). The organic layer was dried and concentrated. Flash chromatography on silica gel (hexane/ethyl acetate, 1:1) gave 2-azido-2-desoxy-3,4,6-tri-*O*-acetyl-D-galactopyranose (900 mg, 71%). This compound (900 mg, 2.70 mmol) was dissolved in dichloromethane (30 mL) and cooled

to 0°C. 1,8-diazabicycloundec-7-ene (DBU) (0.48 mL, 3.26 mmol) and 2,2,2-trifluoro-*N*-phenylacetimidoyl chloride (2.19 mL, 13.6 mmol) were then added and the mixture stirred at 0°C for 30 min. The solvent was then removed and the crude purified by column chromatography (hexane/ethyl acetate, 7:3) to give compound **6** (1.25 g, 92%) as a white solid. HRMS (ESI+): calcd for $C_{20}H_{21}F_3N_4O_8$ (MH^+): 503.1389, found (MH^+): 503.1384.

Synthesis of compound 9. α-Methylserine (**7**) (ref. S3, 1.34 g, 11.2 mmol) was dissolved in H_2O (30 mL) and $NaHCO_3$ (2.50 g, 28.1 mmol) was then added. The resulting mixture was stirred at room temperature until complete dissolution was achieved. The reaction mixture was diluted with acetonitrile (60 mL), followed by the addition of Fmoc-OSu (5.69 g, 16.8 mmol). The white suspension was stirred vigorously at room temperature for 48 h. The acetonitrile was removed under reduced pressure and the aqueous solution was extracted with Et_2O (3×25 mL), followed by acidification and subsequent extraction with a mixture of $CHCl_3$/^{i}PrOH (3:1). The organic layer was concentrated to give compound **8** (2.65 g, 70%), which was used in the next step without further purification. A mixture of dicyclohexylcarbodiimide (2.54 g, 12.1 mmol), *tert*-butanol (1.5 mL, 15.6 mmol) and CuCl (38 mg, 0.38 mmol) was stirred for 5 days. The dark green suspension was then diluted with dichloromethane (20 ml) and compound **8** (1.3 g, 3.81 mmol) in dichloromethane (10 mL) was added dropwise. The reaction was finished within 4 h (hexane/ethyl acetate, 7:3). Precipitated urea was then removed by filtration. The organic layer was washed three times with sat. $NaHCO_3$ solution (30 mL), dried and concentrated. Flash chromatography on silica gel (hexane/ethyl acetate, 7:3) gave compound **9** (1.10 g, 73%). ^{1}H NMR (400 MHz, $CDCl_3$) δ = 1.37-1.57 (m, 12 H, CH_3 MeSer, $C(CH_3)_3$), 2.86 (s, 1H, OH), 3.75- 3.84 (m, 1H, Hβ MeSer), 3.95-4 .01 (m, 1H, Hβ MeSer), 4.15-4.26 (m, 1H, CH_Fmoc), 4.30-4.53 (m, 2H, CH_2 Fmoc), 5.79 (s, 1H, N$\underline{H}$Fmoc), 7.26-7.46 (m, 4H, arom.), 7.54-7.65 (m, 2H, arom.), 7.70-7.81(m, 2H, arom.). ^{13}C NMR (100 MHz, D_2O) δ 20.2 (CH_3 MeSer), 27.9 ($C(\underline{C}H_3)_3$), 47.2 ($\underline{C}$H Fmoc), 60.4 (Cα MeSer), 66.4 (Cβ MeSer), 66.7 ($\underline{C}H_2$ Fmoc), 82.7 ($\underline{C}(CH_3)_3$), 120.0, 125.0, 127.0, 127.1, 127.7, 141.3, 141.3, 143.8, 143.8 (arom.), 155.5, 172.2 (2CO). HRMS (ESI+): calcd for $C_{23}H_{27}NO_5$ (MH+) 398.1962, found (MH+) 398.1967.

Synthesis of compound 10. To a solution of compound **8** (1.30 g, 3.80 mmol) in DMF (8 mL), Cs_2CO_3 (1.48 g, 4.56 mmol) was added at 25°C and the mixture was stirred for 1 h. Next, benzyl bromide (0.68 mL, 5.7 mmol) was added and the reaction mixture stirred at 25°C overnight. The reaction mixture was poured onto a saturated solution of LiBr (50 mL), extracted with ethyl acetate (25 mL), and washed with water (25 mL) and brine (25 mL). The organic layer was dried (Na_2SO_4), filtered and the filtrate was concentrated in vacuum. The residue was purified by silica gel column chromatography (hexane/ethyl acetate, 1:1) to give compound **10** (1.40 g, 85%) as a white solid. ^{1}H NMR (400 MHz, $CDCl_3$) δ = 1.54 (s, 1H, CH_3 MeSer), 3.18 (s, 1H, OH), 3.78-3.96 (m, 1H, Hβ MeSer), 3.96-4.13 (m, 1H, Hβ MeSer), 4.13-4.25 (m, 1H, CH Fmoc), 4.33-4.49 (m, 2H, $C\underline{H}_2$ Fmoc), 5.12-5.32 (m, 2H, $C\underline{H}_2$ Bn), 5.82 (s, 1H, N$\underline{H}$Fmoc), 7.23-7.47 (m, 9H, arom.), 7.53-7.58 (m, 2H, arom.), 7.73-7.83 (m, 2H, arom). ^{13}C NMR (100 MHz, $CDCl_3$) δ 20.3 (CH_3 MeSer), 47.1 (CH Fmoc), 61.4 (Cα MeSer),

66.3 (Cβ MeSer), 66.7 (CH_2 Fmoc), 67.5 (CH_2 Bn), 120.0, 125.0, 127.0, 127.0, 127.0, 127.7, 128.0, 128.1, 128.3, 128.4, 128.5, 135.2, 141.31, 143.7, 143.7 (arom.), 155.6, 172.9 (2CO). HRMS (ESI+): calcd for $C_{26}H_{25}NO_5$ (MH^+): 432.1805, found (MH^+) 432.1811.

Synthesis of compound 11. Compound **9** (570 mg, 1.49 mmol) was stirred with freshly activated powdered 4 Å molecular sieves in toluene (4 mL) and dichloromethane (6 mL) at 25°C for 1 h. Then the mixture was cooled to 0°C and $AgCO_3$ (450 mg, 1.64 mmol) and $AgClO_4$ (40 mg, 0.19 mmol), dissolved in toluene (2 mL), was added dropwise within 30 min. Subsequently, compound **5** (ref. S2, 585 mg, 1.49 mmol) in a mixture of toluene and dichloromethane (16 mL, 1:1) was slowly added to the solution within 15 min. The solution was stirred in the dark under argon at 25 °C for 8h. The suspension was diluted with dichloromethane, filtered through Celite and washed with H_2O (2×50 mL) and a sat. $NaHCO_3$ solution (50 mL). After drying of the organic phase, the solvent was evaporated and the residue was purified by column chromatography (a first column in toluene/ethyl acetate, 7:3, and a second column in hexane/ethyl acetate, 6.5:3.5) to give compound **11** (500 mg, 48%) as a colorless oil. ^1H NMR (400 MHz, $CDCl_3$) δ = 1.43-1.56 (m, 12 H, CH_3 MeSer, $C(CH_3)_3$), 1.95 (s, 3H, $COCH_3$), 2.05 (s, 3H, $COCH_3$), 2.13 (s, 3H, $COCH_3$), 3.58-366 (m, 1H, H2), 3.91 (d, 1H, *J* = 9.2 Hz, Hβ MeSer), 3.96-4.10 (m, 2H, H6), 4.11-4.19 (m, 1H, H5), 4.18-4.26 (m, 1H, CH Fmoc), 4.26-4.46 (m, 3H, Hβ MeSer, CH_2 Fmoc), 4.89-4.97 (m,1H, H1), 5.24-5.29 (m, 1H, H3), 5.38-5.43 (m, 1H, H4), 5.89 (s, 1H, NH Fmoc), 7.29-7.44 (m, 4H, arom.), 7.60-7.66 (m, 2H, arom.), 7.73-7.80 (m, 2H, arom.). ^{13}C NMR (100 MHz, $CDCl_3$) δ 20.5, 20.6, 20.6, 20.6 (CH_3 MeSer, 3 $CO\underline{C}H_3$), 27.8 ($C(\underline{C}H_3)_3$), 47.16 (CH Fmoc), 57.6 (C2), 60.2 (Cα MeSer), 61.3 (C6), 66.6, 66.9 (C5, CH_2 Fmoc), 67.4 (C4), 68.0 (C3), 82.9 $\underline{C}(CH_3)_3$), 98.9 (C1), 119.9, 125.1, 127.1, 127.6, 141.3, 143.9, 144.0 (arom.), 154.7, 169.7, 170.0, 170.3, 171.2 (5CO). HRMS (ESI+): calcd for $C_{35}H_{42}N_4O_{12}$ (MH^+): 711.2872, found (MH^+) 711.2862.

Synthesis of compound 12. To a solution of compound **11** (500 mg, 0.67 mmol) in THF/AcOH/Ac_2O (15 mL, 3:2:1) cooled to 0°C, Zn (264 mg, 4.05 mmol) and 1 mL of a saturated $CuSO_4$ solution were added. The mixture was stirred for 1 h. After filtration, the solvent mixture was removed in vacuum and the residue was purified by a silica gel column chromatography (ethyl acetate/hexane, 7:3) to give compound **12** (420 mg, 82%) as a white solid. ^1H NMR (400 MHz, $CDCl_3$) δ = 1.36-1.47 (m, 12 H, CH_3 MeSer, $C(CH_3)_3$), 1.84 (s, 3H, $COCH_3$), 1.86 (s, 3H, $COCH_3$), 1.92 (s, 3H, $COCH_3$), 2.07 (s, 3H, $COCH_3$), 3.63-3.72 (m, 1H, Hβ MeSer), 3.89-3.95 (dd, 1H, *J* = 9.4, 5.0 Hz, H6), 4.08 (m, 2H, H6, H5), 4.08-4.14 (m, 1H, Hβ MeSer), 4.18 (t, 1H, *J* = 6.7 Hz, CH Fmoc), 4.28-4.37 (m, 2H, CH_2 Fmoc), 4.42-4.56 (m, 1H, H2), 4.77-4.84 (m, 1H, H1), 4.79-5.04 (m, 1H, H3), 5.23-5.28 (m, 1H, H4), 5.69 (m, 1H, NHFmoc), 5.72-5.80 (m, 1H, NHAc), 7.21-7.38 (m, 4H, arom.), 7.53-7.59 (m, 2H, arom.), 7.67-7.72 (m, 2H, arom.). ^{13}C NMR (100 MHz, $CDCl_3$) δ 19.5, 19.5, 19.7, 19.7 (CH_3 MeSer, 3 $CO\underline{C}H_3$), 20.0 ($CO\underline{C}H_3$), 22.2 ($CO\underline{C}H_3$), 26.9 ($C(\underline{C}H_3)_3$), 46.1 (CH Fmoc), 46.6 (C2), 59.1 (Cβ MeSer), 59.4 (Cα MeSer), 60.5 (C6), 65.7 (CH_2 Fmoc), 66.1, 66.2 (C4, C5), 67.5 (C3), 69.6 (Cβ MeSer), 81.9 ($C(CH_3)_3$), 97.4 (C1), 119.0, 124.0, 126.1, 126.1, 126.7, 127.2, 128.0, 140.3,

142.8, 142.9 (arom.), 153.8, 169.0, 169.3, 169.3, 169.8, 170.8 (6 CO). HRMS (ESI+): calcd for $C_{37}H_{46}N_2O_{13}$ (MH+): 727.3073, found (MH+) 727.3062.

Synthesis of compound 13. Compound **10** (450 mg, 1.04 mmol) and compound **6** (602 mg, 1.20 mmol) were dissolved in Et_2O (15 mL) under an argon atmosphere in the presence of molecular sieves. The reaction mixture was stirred at 25°C for 30 min and, after cooling to -40 °C, trifluoromethanesulfonic acid (9.26 µL, 115 µmol) was added. The reaction was stirred for an additional 15 min., the molecular sieves were then filtered off and the solvent was removed by evaporation. The residue was purified by a silica gel column chromatography (toluene/ethyl acetate, 7:3) to give the α anomer **13** (550 mg, 70%) as a white solid. ^1H NMR (400 MHz, $CDCl_3$) δ = 1.63 (s, 1H, CH_3 MeSer), 1.97 (s, 3H, $COCH_3$), 2.10 (s, 3H, $COCH_3$), 2.17 (s, 3H, $COCH_3$), 3.56-3.64 (m, 1H, H2), 3.91-4.17 (m, 4H, Hβ MeSer, H6, H5), 4.26 (t, 1H, J = 6.9 Hz, CH Fmoc), 4.30-4.50 (m, 3H, Hβ MeSer,CH_2 Fmoc), 4.89-4.96 (m, 1H, H1), 5.20-5.36 (m, 3H, CH_2 Bn, H3), 5.39-5.43 (m, 1H, H4), 5.90 (s, 1H, NHFmoc), 7.29-7.47 (m, 9H, arom.), 7.61-7.68 (m, 2H, arom.), 7.78-7.83 (m, 2H, arom.). ^{13}C NMR (100 MHz, $CDCl_3$) δ 20.5, 20.6, 20.6, 20.6 (CH_3 MeSer, 3 CO<u>C</u>H_3), 47.1 (CH Fmoc), 57.4 (C2), 60.0 (Cα MeSer), 61.3 (C6), 66.8 (C5,), 67.0 (CH_2 Fmoc), 67.4, 67.9, 67.9 (CH_2 Bn, C4, C3), 70.57 (Cβ MeSer) , 98.8 (C1), 120.0, 125.1, 127.1, 127.7, 128.3, 128.5, 128.6, 135.1, 141.3, 143.8, 143.9 (arom.), 154.7, 169.7, 170.0, 170.4, 172.0 (5 CO). HRMS (ESI+): calcd for $C_{38}H_{40}N_4O_{12}$ (MH^+): 745.2715, found (MH^+) 745.2714.

Synthesis of compound 14. To a solution of compound **13** (232 mg, 0.31 mmol) in THF/AcOH/Ac_2O (7 mL, 3:2:1) cooled to 0 °C, Zn (129 mg, 2.00 mmol) and 1 mL of a saturated $CuSO_4$ solution were added. The mixture was stirred for 1 h. After filtration, the solvent mixture was removed in vacuum and the residue was purified by a silica gel column chromatography (ethyl acetate/hexane, 7:3) to give compound **14** (220 mg, 92%) as a white solid. ^1H NMR (400 MHz, $CDCl_3$) δ = 1.57 (s, 1H, CH_3 MeSer), 1.93 (s, 3H, $COCH_3$), 2.00 (s, 3H, $COCH_3$), 2.03 (s, 3H, $COCH_3$), 2.18 (s, 3H, $COCH_3$), 3.77-3.92 (m, 1H, Hβ MeSer), 3.97-4.20 (m, 4H, Hβ MeSer, H6, H5), 4.26 (t, 1H, J = 6.5 Hz, CH Fmoc), 4.40-4. 50 (m, 2H, CH_2 Fmoc), 4.51-4.62 (m, 1H, H2), 4.83-4.92 (m,1H, H1), 5.03-5.11 (m, 1H, H3), 5.12-5.27 (m, 2H, CH_2 Bn), 5.31-5.36 (m, 1H, H4), 5.70-5.78 (m, 1H, NHAc), 5.84 (s, 1H, NHFmoc), 7.26-7.50 (m, 9H, arom.), 7.61-7.67 (m, 2H, arom.), 7.77-7.82 (m, 2H, arom.). ^{13}C NMR (100 MHz, $CDCl_3$) δ 20.5, 20.6, 20.7, 20.8 (CH_3 MeSer, 3 $COCH_3$), 23.2 ($COCH_3$), 47.0 (CH Fmoc), 47.6 (C2), 60.1 (Cα MeSer), 61.7 (C6), 66.8, 67.1, 67.1 (CH_2 Fmoc, C4, C5), 67.7 (CH_2 Bn), 68.3 (C3), 71.4 (Cβ MeSer), 98.8 (C1), 120.0, 124.9, 127.1, 127.7, 128.2, 128.7, 128.8, 134.8, 141.3, 143.8, 143.8 (arom.), 154.9, 170.1, 170.3, 170.4, 170.8, 172.1 (6 CO). HRMS (ESI+): calcd for $C_{40}H_{44}N_2O_{13}$ (MH^+): 761.2916, found (MH^+) 761.2930.

Synthesis of compound 2. *Route A:* Compound **12** (420 mg, 0.58 mmol) was dissolved in dichloromethane/TFA (1:1, 6 mL). After stirring the mixture for 2 h at 25 °C, the solvent was removed and the crude product was purified by silica gel column chromatography (CH_2Cl_2/MeOH, 9:1, 0.1% CH_3CO_2H) to give building block **2** (360 mg, 93%) as a white solid.

Route B: A solution of compound **14** (220 mg, 0.30 mmol) in MeOH (10 mL) was treated with 10% Pd/C (44 mg) as a catalyst. The reaction mixture was shaken under an atmosphere of H_2 for 1 h at 25°C. The catalyst and the solvent were removed and the crude product was purified by silica gel column chromatography (CH_2Cl_2/MeOH, 9:1, 0.1% CH_3CO_2H) to give building block **2** (180 mg, 89%) as a white solid. HRMS (ESI+): calcd for $C_{33}H_{38}N_2O_{13}$ (MH^+): 671.2449, found (MH^+) 671.2440.

Molecular dynamics (MD) simulations. Parameters for Pam_3-Cys-OH derivative were generated with the *antechamber* module of Amber12 (ref S4) using the general Amber force field (GAFF),[S5] with partial charges set to fit the electrostatic potential generated with HF/6-31G(d) by RESP.[S6] The charges are calculated according to the Merz-Singh-Kollman scheme using Gaussian 09 (ref S7). The lipoglycopeptides were immersed in micelle consisting of 65 DPC (dodecylphosphocholine) lipids.[S8] The lipid14 (ref S9) and GAFF force fields were used to properly reproduce the conformational behaviour of the micelles. The *ff14SB* (ref S10) and Gycam06j (ref S11) force fields were employ to properly model the glycopeptide fragment. The liposome, together with the corresponding lipoglycopeptide, was immersed in a water box with a 10 Å buffer of TIP3P[S12] water molecules. A two-stage geometry optimization approach was performed. The first stage minimizes only the positions of solvent molecules and the second stage is an unrestrained minimization of all the atoms in the simulation cell. The systems were then gently heated by incrementing the temperature from 0 to 300 K under a constant pressure of 1 atm and periodic boundary conditions. Harmonic restraints of 30 kcal·mol^{-1} were applied to the solute, and the Andersen temperature coupling scheme[S13] was used to control and equalize the temperature. The time step was kept at 1 fs during the heating stages, allowing potential inhomogeneities to self-adjust. Water molecules are treated with the SHAKE algorithm such that the angle between the hydrogen atoms is kept fixed. Long-range electrostatic effects are modelled using the particle-mesh-Ewald method.[S14] An 8 Å cutoff was applied to Lennard-Jones and electrostatic interactions. Each system was equilibrated for 2 ns with a 2 fs time step at a constant volume and temperature of 300 K. Production trajectories were then run for additional 100 ns under the same simulation conditions.

Molecular dynamics (MD) simulations with time averaged restraints (MD-tar). The simulations were carried out with AMBER 12 package with ff14SB and GLYCAM06j force fields. Prior to MD-tar productive simulations, we performed an equilibration protocol consisting of an initial minimization of the water box of 5000 steps, followed by a 2500-step minimization of the whole system. Then, the TIP3P water box was heated at constant volume until 298 K using a time constant for the heat bath coupling of 1 ps. The equilibration finished with 200 ps of MD simulation without restraints, at a constant pressure of 1 bar and turning on the Langevin temperature scaling with a collision frequency of 1 ps. Furthermore, non- bonded interactions were cut off at 8.0 Å and updated every 25 steps. Periodic boundary conditions and the Particle Mesh Ewald method were turned on in every step of the equilibration protocol to evaluate the long-range electrostatic forces, using a grid spacing of approximately 1 Å. The NOE-derived distances shown in the Supporting Information were imposed as time-averaged constraint, applying

an r^{-6} averaging. The equilibrium distance range was set to r_{exp} − 0.2 Å ≤ r_{exp} ≤ r_{exp} + 0.2 Å. Trajectories were run at 298 K, with a decay constant of 2000 ps and a time step of 1 fs. The force constants r_{k2} and r_{k3} used in each case were 10 kcal·mol^{-1}·Å^{-2}. The overall simulation length for the simulations was 20 ns. The coordinates were saved each 1 ps, thus, obtaining MD trajectories of 20000 frames each. A convergence within the equilibrium distance range was obtained in the simulations. The analysis of the MD-tar trajectories has been carried out with the *cpptraj* modules of AMBER 12.

Degradation studies with human plasma.

Commercially available serum from human male AB plasma was used. Glycopeptides APD**Thr**(α-D-GalNAc)RP and APD**MeSer**(α-D-GalNAc)RP were suspended in a solution of 20% human serum in water at 37°C. Degradation of the peptides was determined every hour by using UPLC-micrOTOF-Q (column: ACQUITY UPLC BEH C18 1,7; diameter: 2.1 mm and length: 100 mm), eluting a mixture of 5 μL of the glycopeptide-serum solution in 795 μL of water and employing an isocratic method (70% H_2O, 30% acetonitrile, temperature: 40°C).

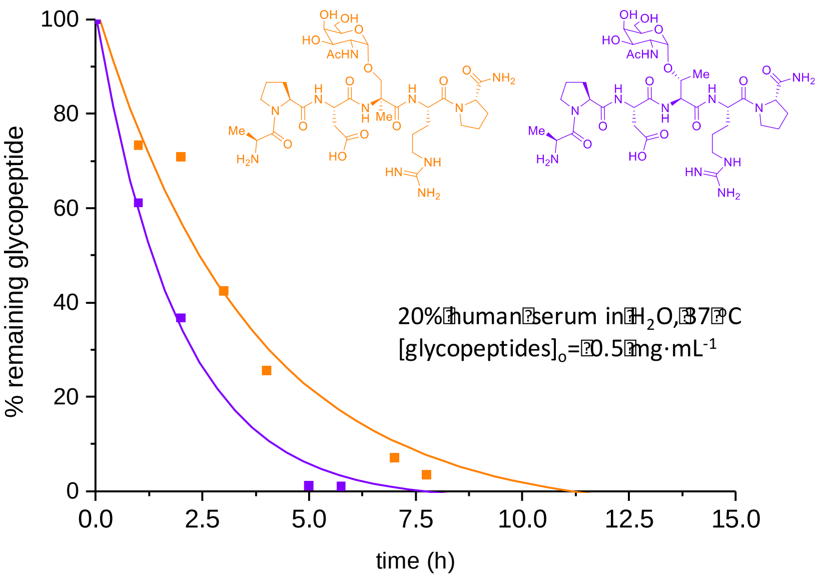

Figure S1

Liposome preparation for immunizations. Glycolipopeptide **1** was incorporated into phospholipid-based small unilamellar vesicles (SUVs) by hydration of a thin film of the synthetic compound **1**, egg phosphatidylglycerol, cholesterol and egg phosphatidylcholine at the ratio 1:2.5:5:6.5, respectively in a HEPES buffer (10 mM, pH 7.4) containing NaCl (145 mM) followed by extrusion through a 0.1 μm Nucleopore® polycarbonate membrane.

Immunizations. Eight to 12-week-old MUC1.Tg mice (C57BL/6; H-2b) that express human MUC1 at physiological level were immunized four-times at biweekly intervals at the base of the tail intradermally with liposomal preparations of the vaccine construct (25 μg containing 3 μg of carbohydrate) or with empty liposomes. Endpoint was one week after 4th immunization.

Serologic assays. Anti-MUC1 IgG, IgG1, IgG2a, IgG2b, IgG3 and IgM antibody titers were determined by enzyme-linked immunosorbent assay (ELISA). Briefly, ELISA plates were coated with a conjugate of the MUC1 glycopeptide or peptide conjugated to BSA through a maleimide linker (BSA-MI-CTSAPDT(α-D-GalNAc)RPAP BSA-MI-CTSAPDTRPAP, respectively. MI stands for maleimide). Serial dilutions of the sera were allowed to bind to immobilized MUC1. Detection was accomplished by the addition of phosphate-conjugated anti-mouse antibodies and *p*-nitrophenyl phosphate. To determine antibody titers against the T_{helper} (polio) epitope, Reacti-bind NeutrAvidin coated and pre-blocked plates were incubated with biotin-labeled T_{helper} (10 μg/mL; 100 μL/well) for 2 h. Next, serial dilutions of the sera were allowed to bind to immobilized T_{helper} epitope. Detection was accomplished as described above. The antibody titer was defined as the highest dilution yielding an optical density of 0.1 or greater over that of normal control mouse sera.

Cell culture. Human breast adenocarcinoma cells MCF7[S16] (obtained from ATCC) were cultured in Eagle's minimum essential medium with L-glutamine (2 mM) and Earle's BSS, modified to contain sodium bicarbonate (1.5 g/L), non-essential amino acids (0.1 mM) and sodium pyruvate (1 mM) and supplemented with bovine insulin (0.01 mg/mL; Sigma), penicillin (100 U/mL), streptomycin (0.1 μg/mL) and FBS (10%). C57mg.MUC1 mammary gland tumor cells[S17] (a kind gift of Dr. S. Gendler) were cultured in DMEM supplemented with penicillin (100 U/mL), streptomycin (0.1 μg/mL), L-glutamax (2 mm), FCS (10%) and G418 (150 μg/mL). All cells were maintained in a humid 5% CO_2 atmosphere at 37°C.

Cell recognition analysis by fluorescence measurements. Pre- and post-immunization sera were diluted 50-fold and incubated with MCF7 or C57mg.MUC1 cell suspensions for 30 min on ice. Next, the cells were washed and incubated with goat anti-mouse IgG γ-chain specific antibody conjugated to FITC (Sigma) for 20 min on ice. Following three washes, cells were lysed in passive lysis buffer (Promega). Cell lysates were analyzed for fluorescence intensity (485 ex / 520 em) using a microplate reader (BMG Labtech). Data points were collected in triplicate.

Statistical analysis. Comparisons were performed using unpaired t test with equal SD. Differences were considered significant when $P < 0.05$.

MALDI-TOF spectrum for **compound 1**

HRMS (ESI+): calcd for $C_{217}H_{367}N_{45}O_{53}S_2Na$ (MH^+) 4538.6745, found (MH^+) 4538.5.

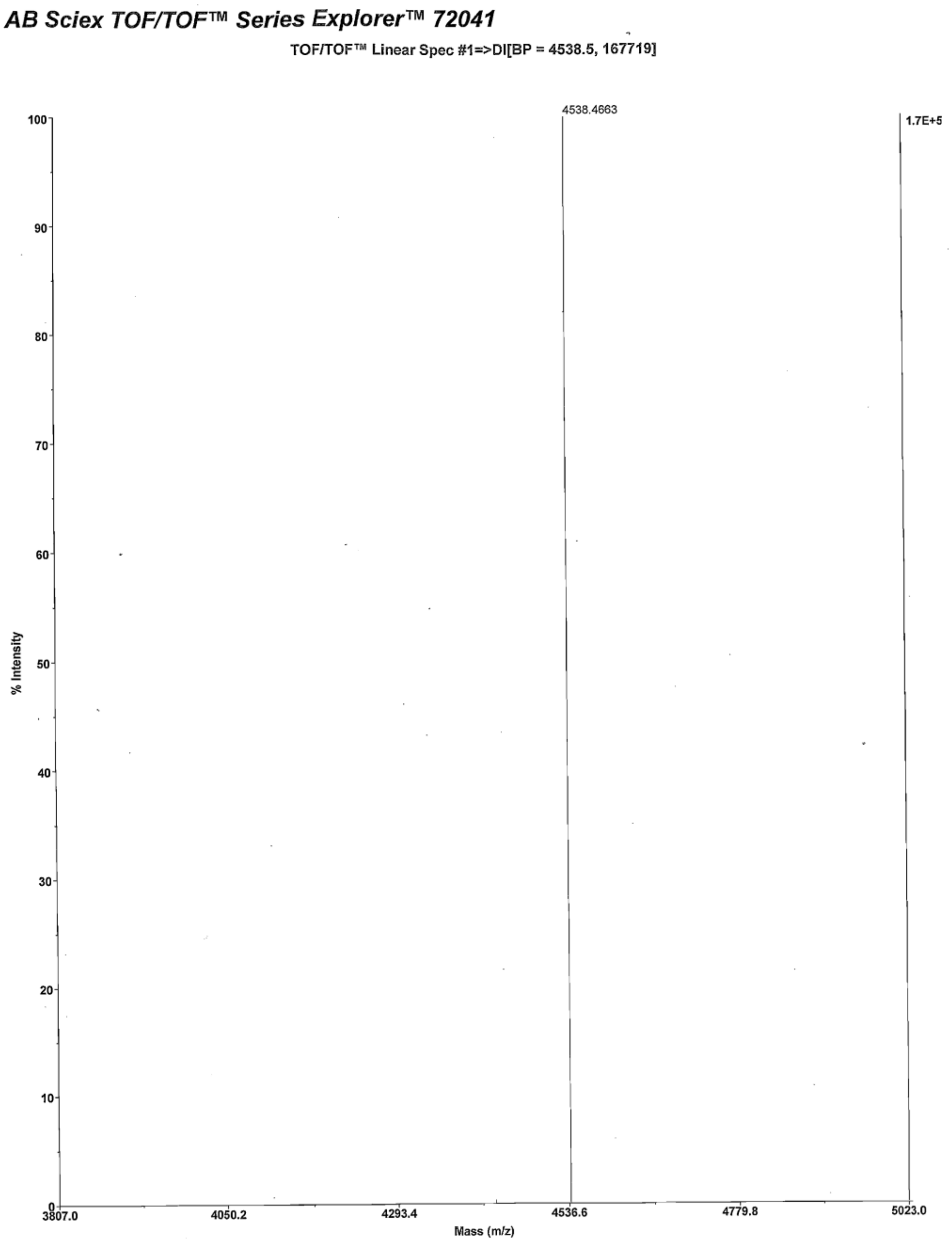

Figure S2

Synthesis of compound g1. This compound was synthesized following the SPPS methodology.

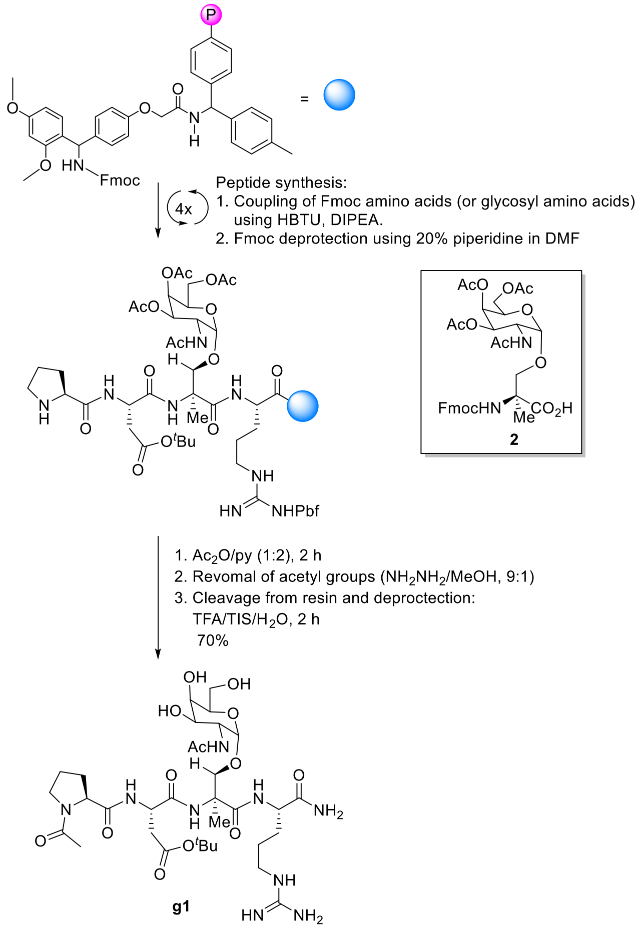

Scheme 1. Synthesis of glycopeptide **g1**. Building block **2** (main text) was manually incorporated to the peptide sequence. HBTU=1*H*-benzotriazol-1-yl)-*N*,*N*,*N'*,*N'*-tetramethyluronium hexafluorophosphate. DIPEA = Diisopropylethylamine. TFA=trifluoroacetic acid. TIS=triisopropylsilane. DMF=dimethylformamide.

^{1}H NMR 400 MHz in D_2O registered at 298K for **compound g1**

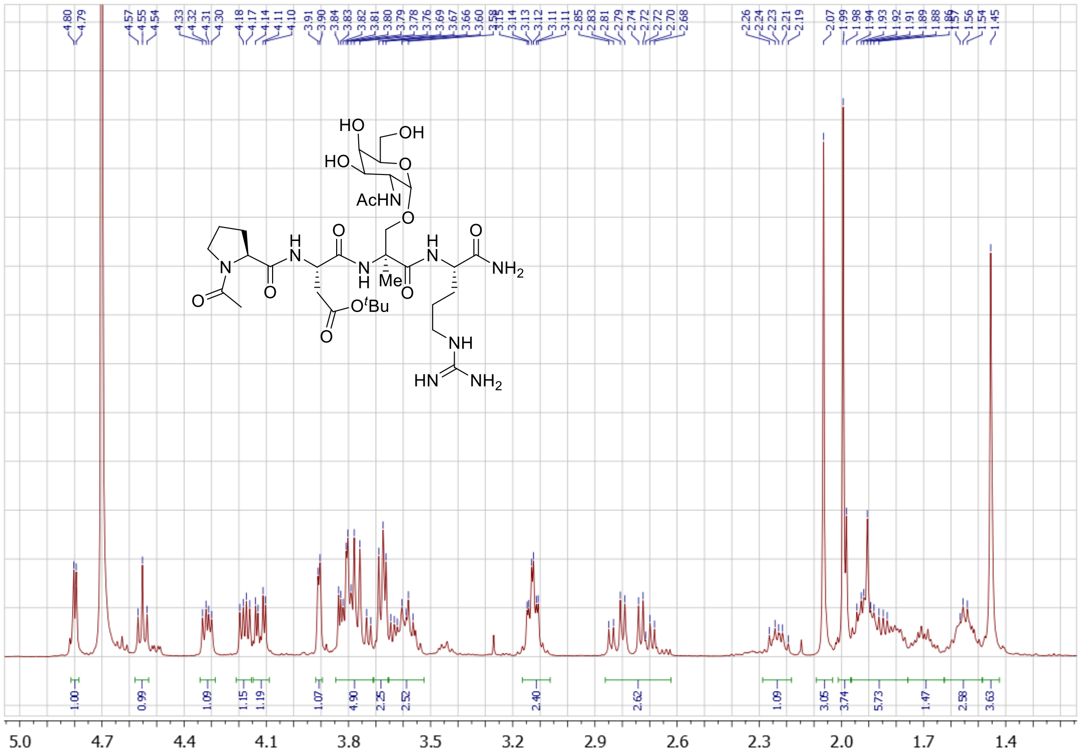

Duplication of some signals in low percentage is observed due to the existence of cis-trans proline rotamers.

^{13}C NMR 100 MHz in D_2O registered at 298K for **compound g1**

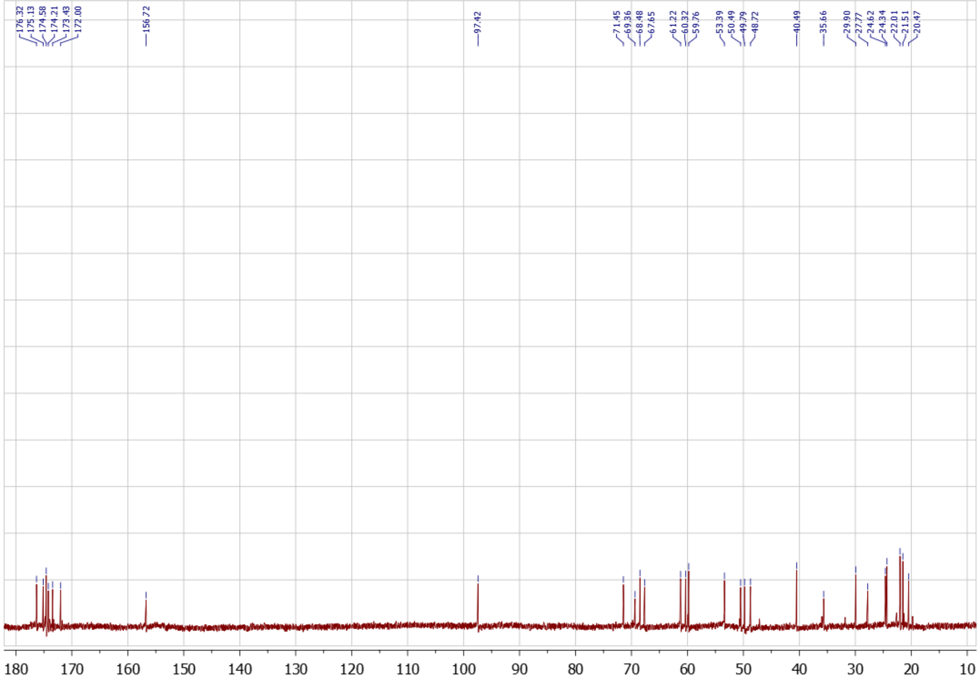

COSY in D_2O registered at 298K for **compound g1**

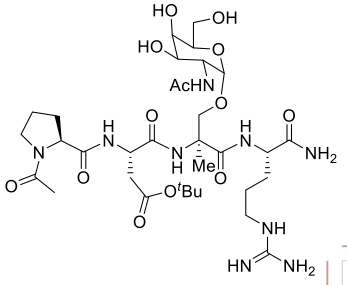

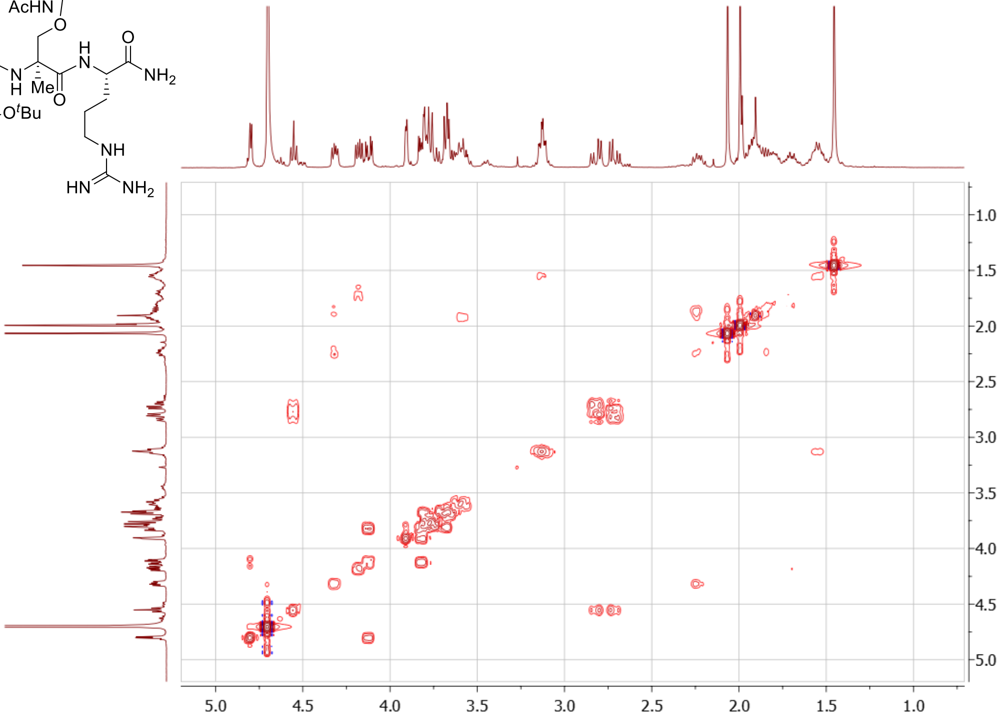

HSQC in D_2O registered at 298K for **compound g1**

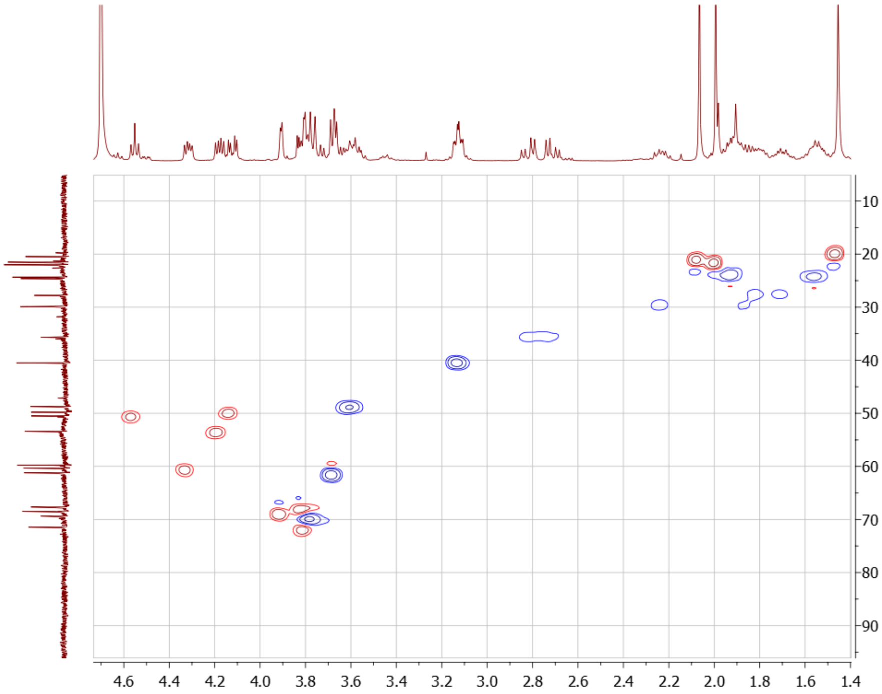

^{1}H NMR 400 MHz in D_2O registered at 298K for **compound g2**

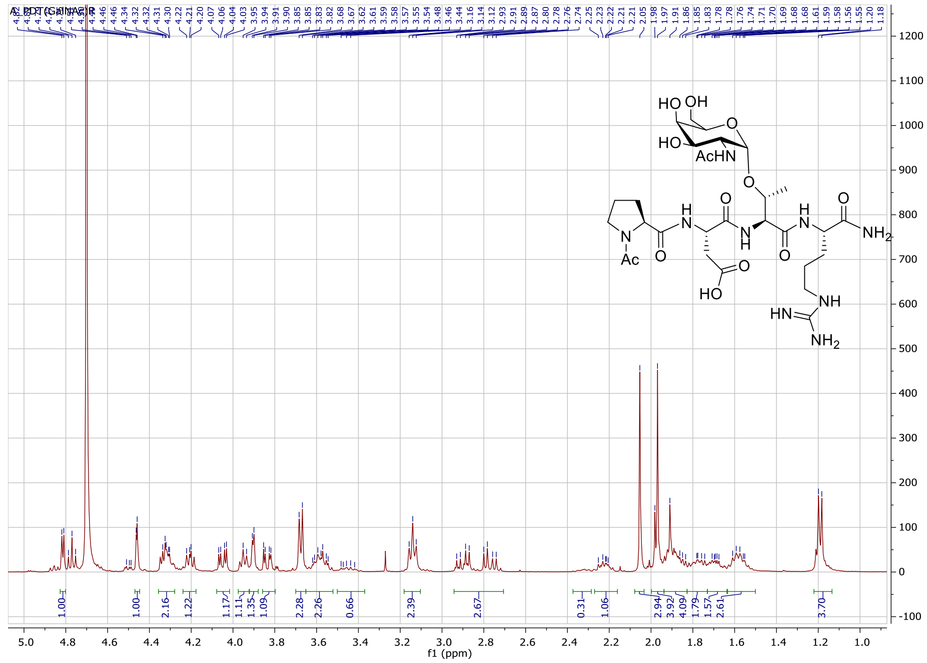

Duplication of some signals in low percentage is observed due to the existence of cis-trans proline rotamers.

^{13}C NMR 100 MHz in D_2O registered at 298K for **compound g2**

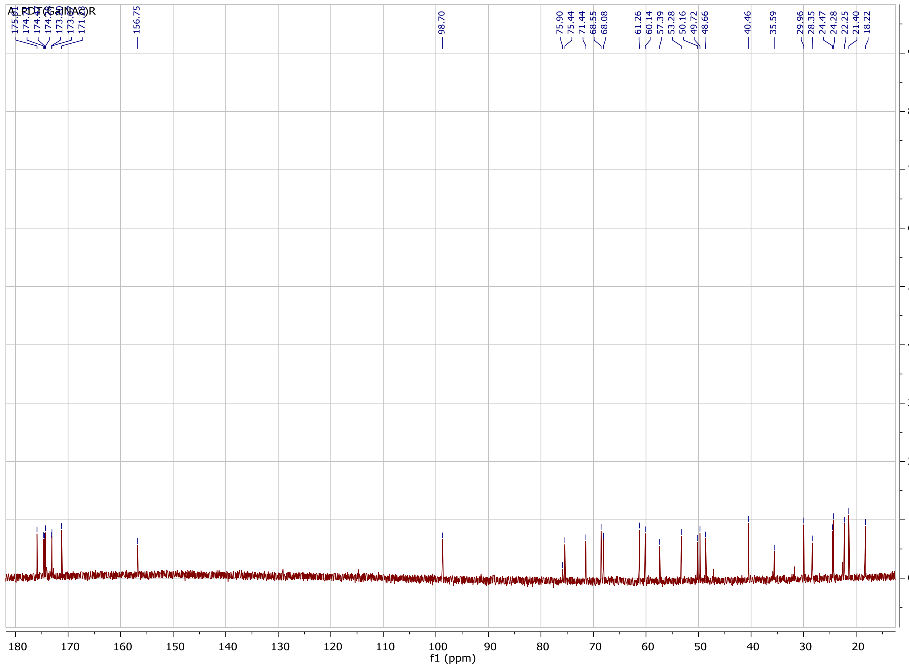

COSY in D_2O registered at 298K for **compound g2**

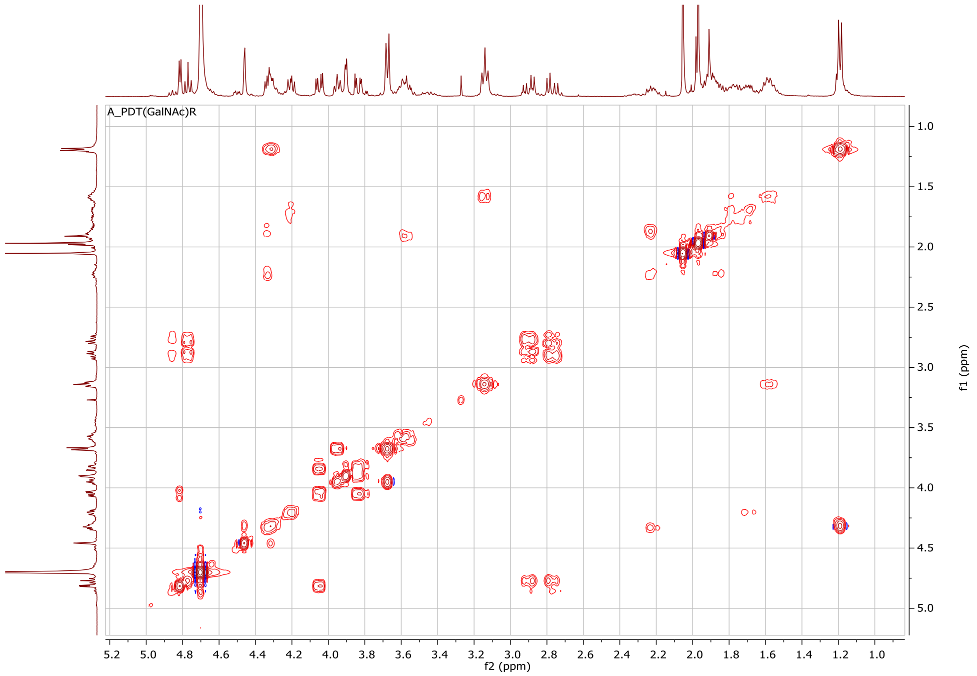

HSQC in D_2O registered at 298K for **compound g2**

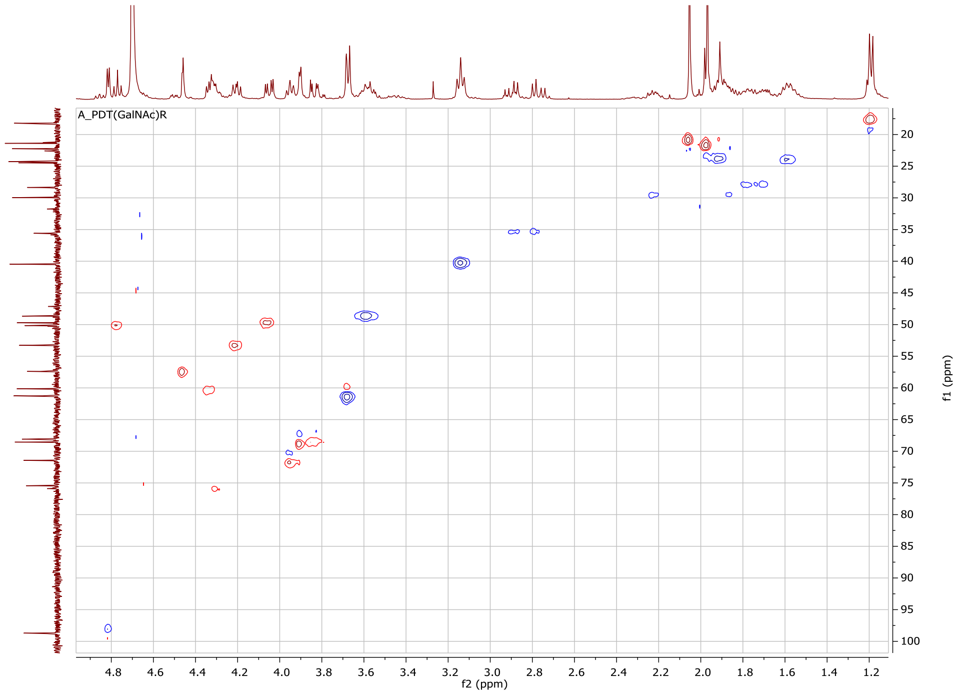

^{1}H NMR 400 MHz in $CDCl_3$ registered at 298K for **compound 2**

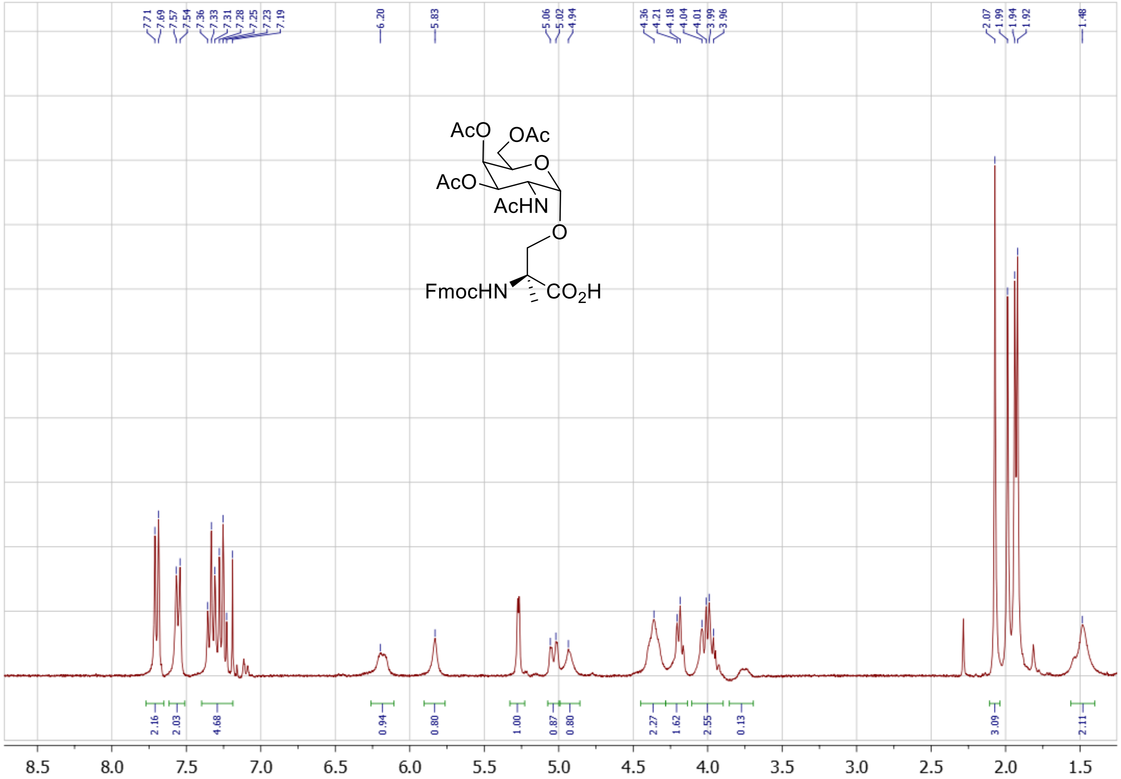

^{13}C NMR 100 MHz in $CDCl_3$ registered at 298K for **compound 2**

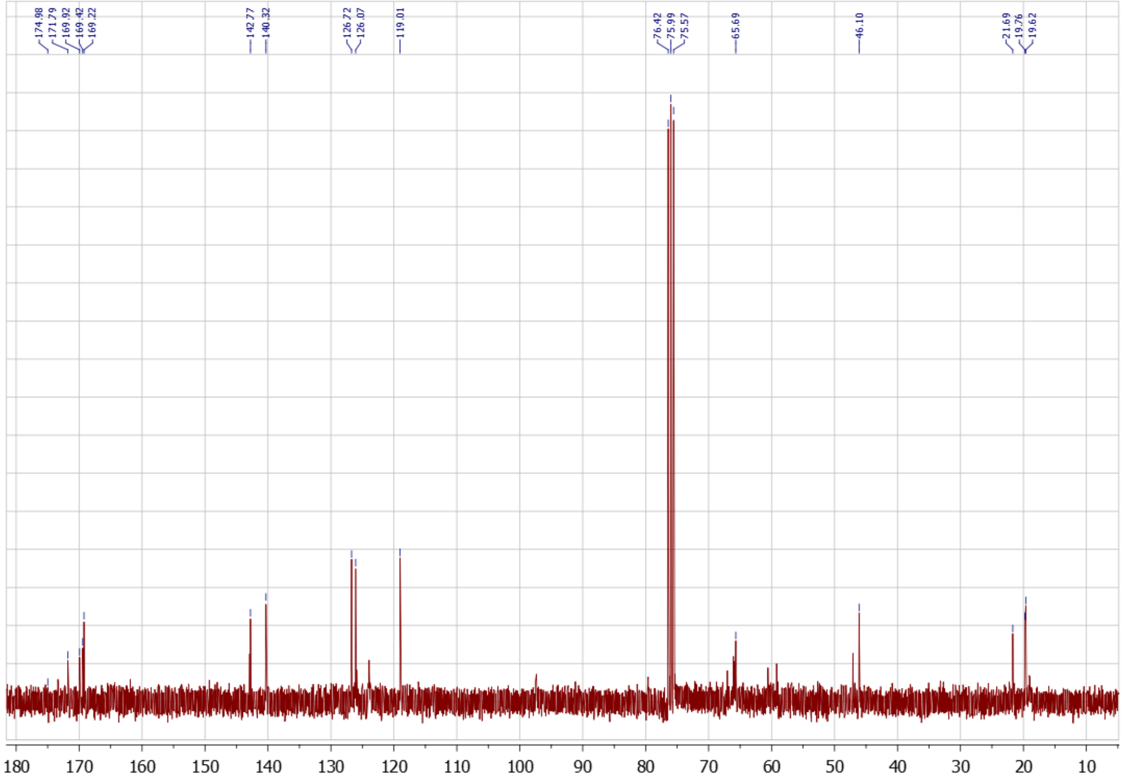

COSY in $CDCl_3$ registered at 298K for **compound 2**

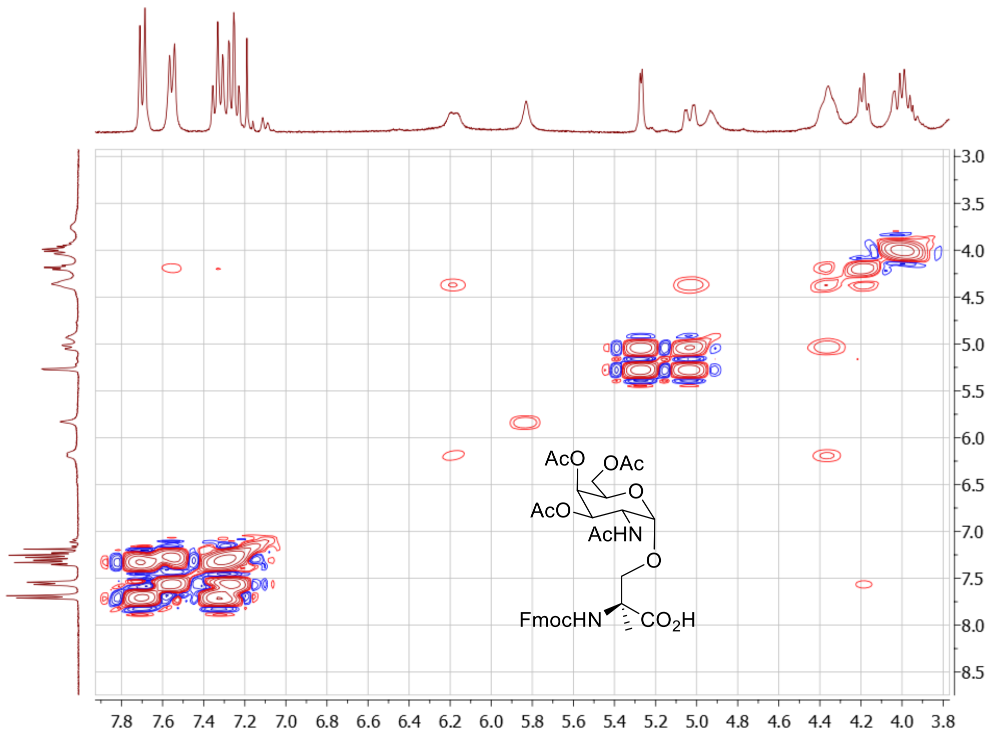

HSQC in $CDCl_3$ registered at 298K for **compound 2**

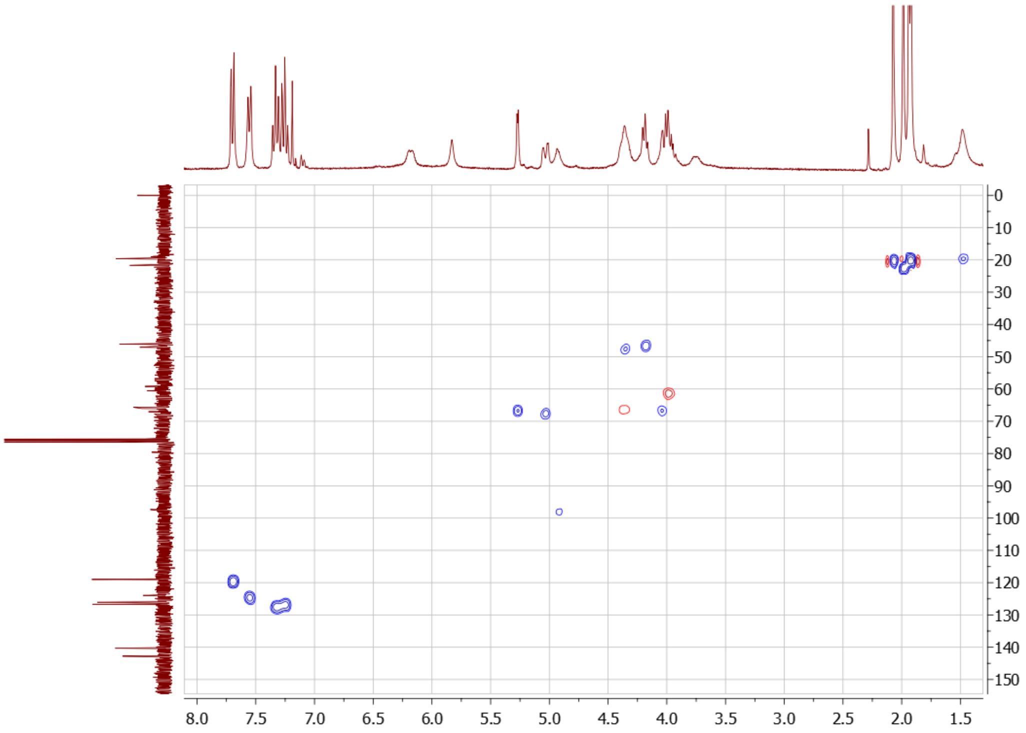

^{1}H NMR 400 MHz in $CDCl_3$ registered at 298K for **compound 9**

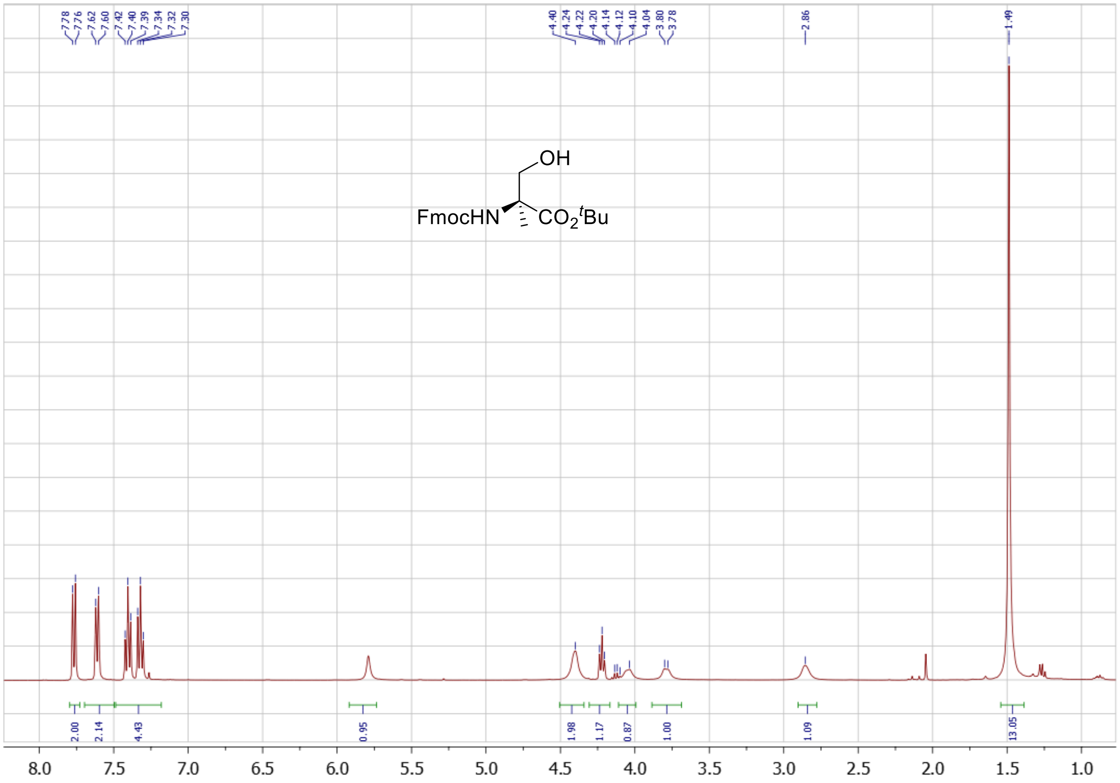

^{13}C NMR 100 MHz in $CDCl_3$ registered at 298K for **compound 9**

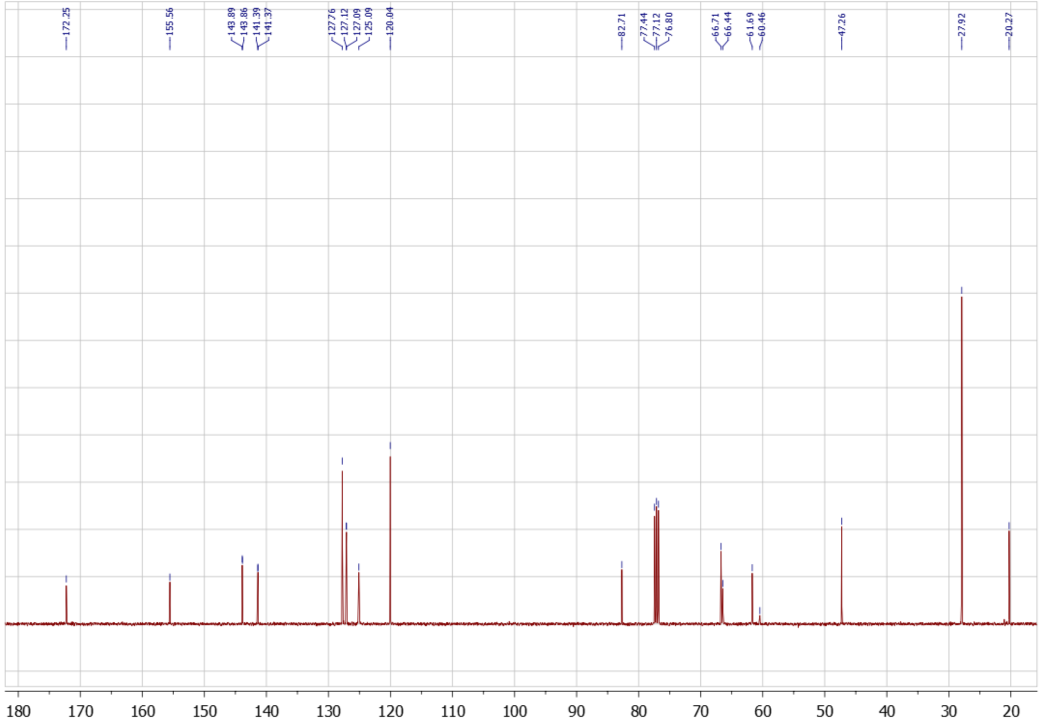

COSY in $CDCl_3$ registered at 298K for **compound 9**

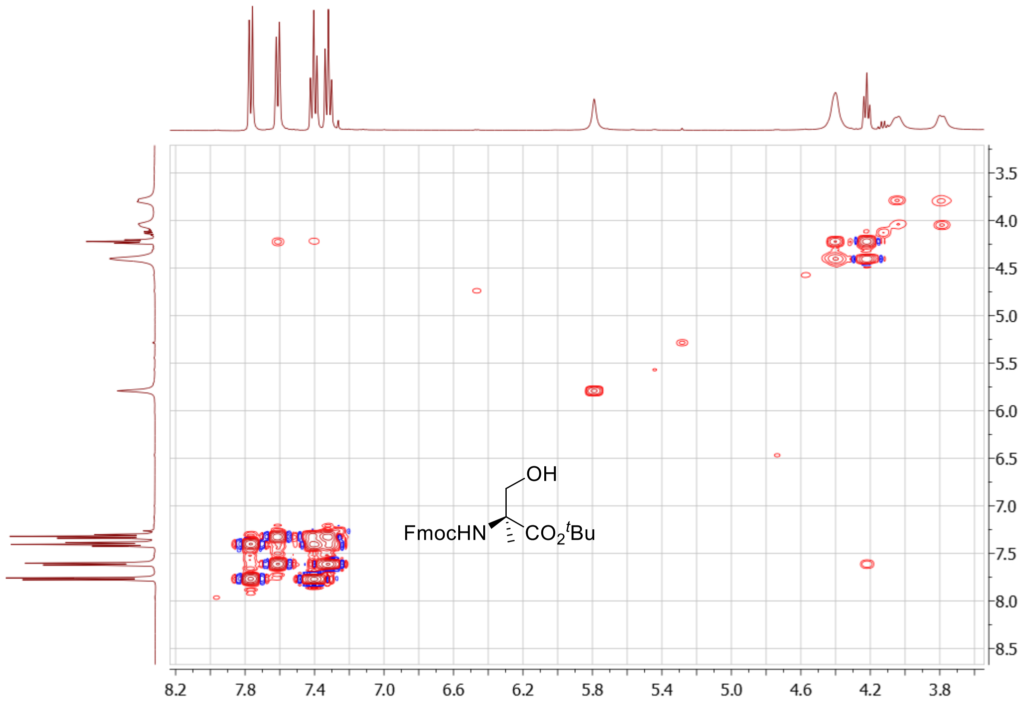

HSQC in $CDCl_3$ registered at 298K for **compound 9**

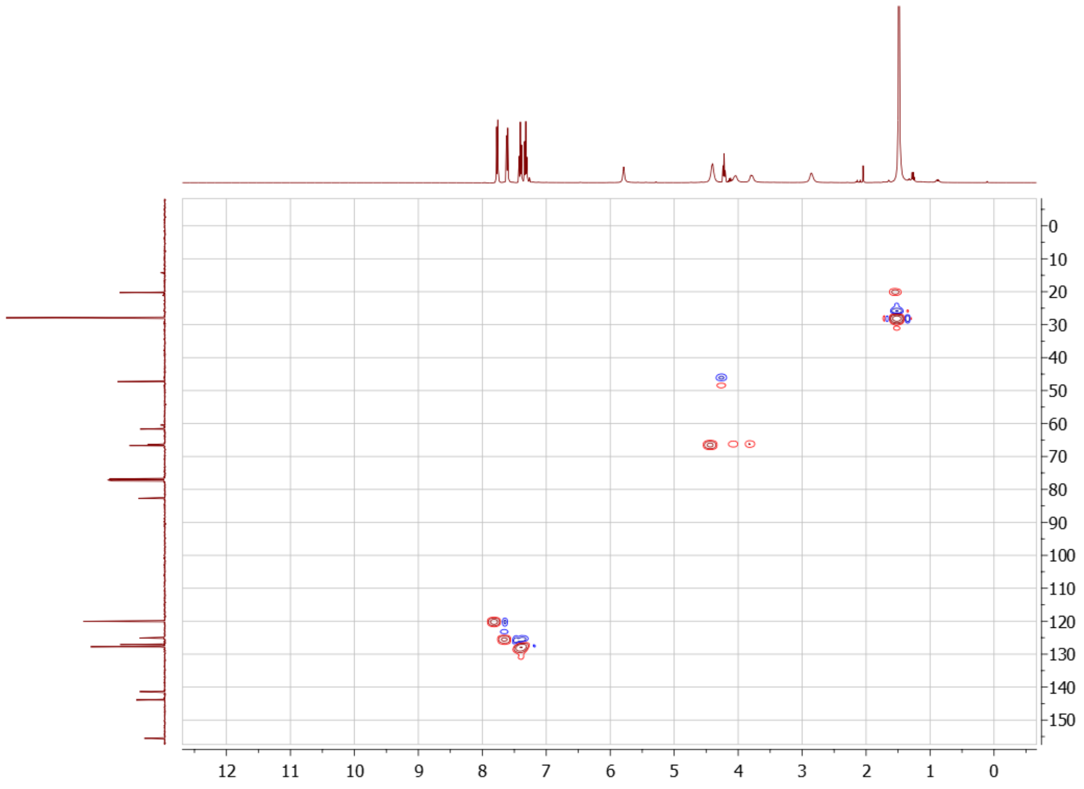

^{1}H NMR 400 MHz in $CDCl_3$ registered at 298K for **compound 10**

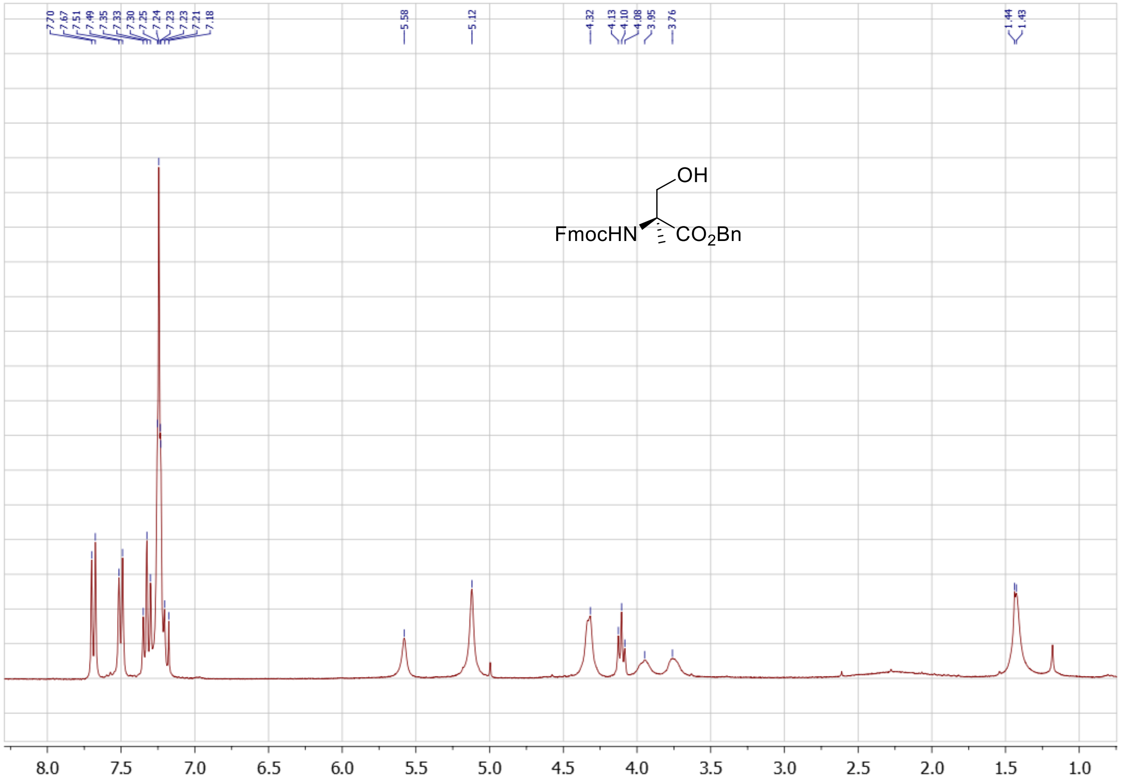

^{13}C NMR 100 MHz in $CDCl_3$ registered at 298K for **compound 10**

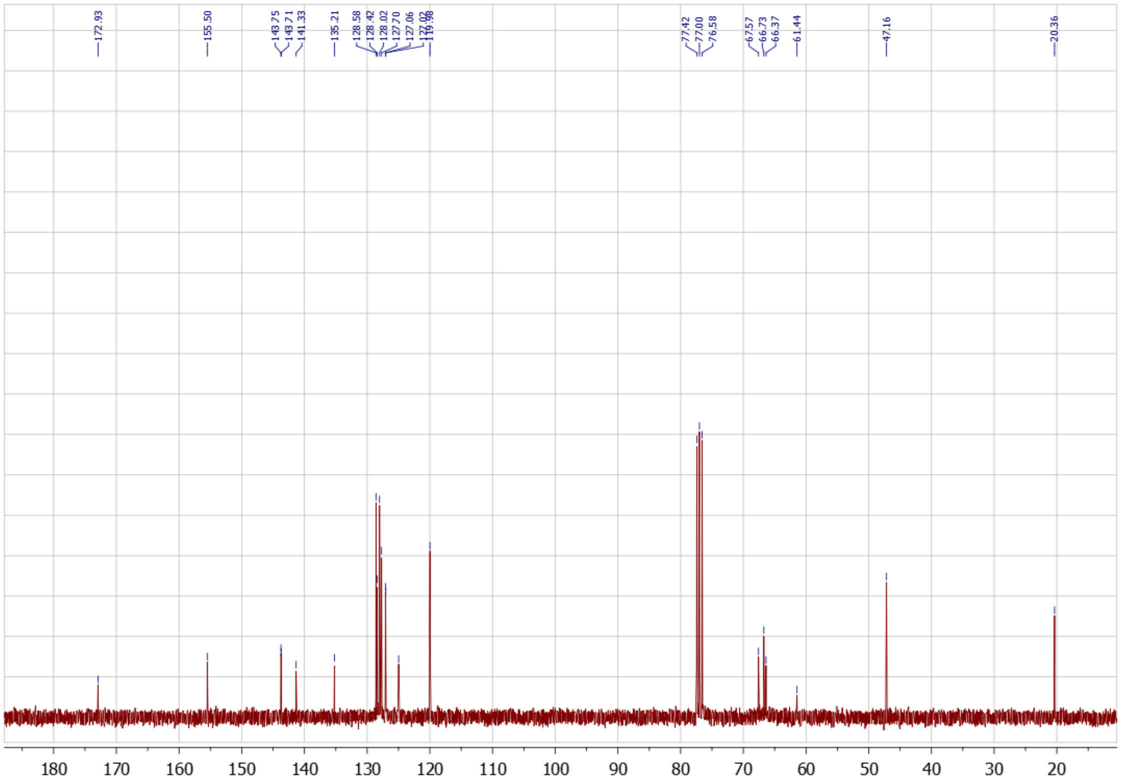

COSY in $CDCl_3$ registered at 298K for **compound 10**

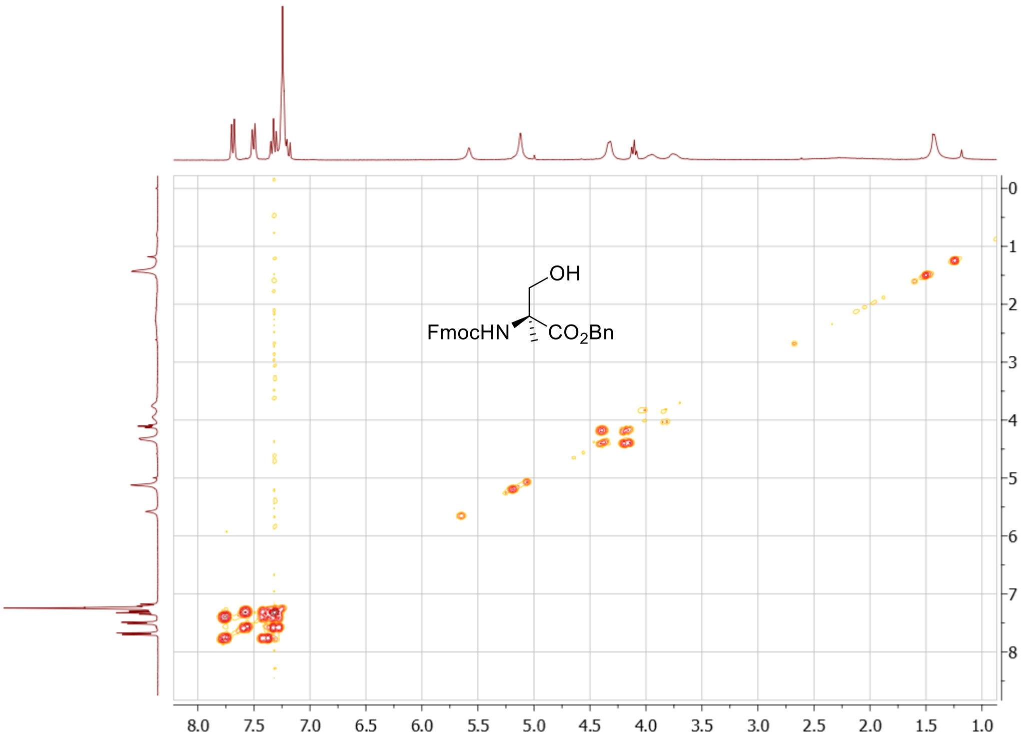

HSQC in $CDCl_3$ registered at 298K for **compound 10**

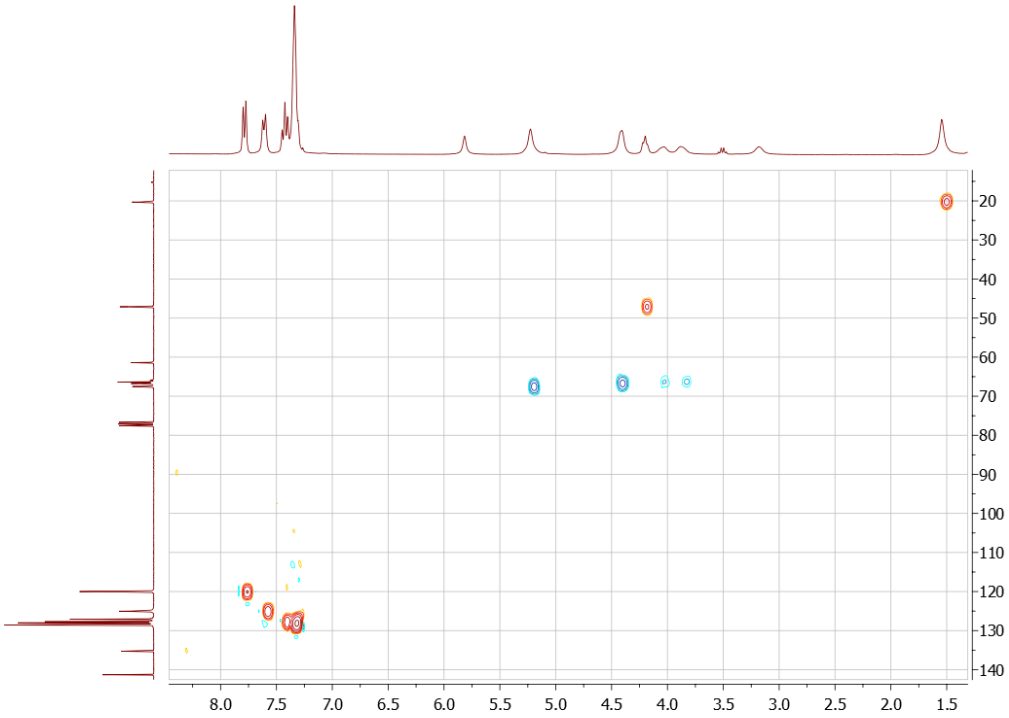

^{1}H NMR 400 MHz in $CDCl_3$ registered at 298K for **compound 11**

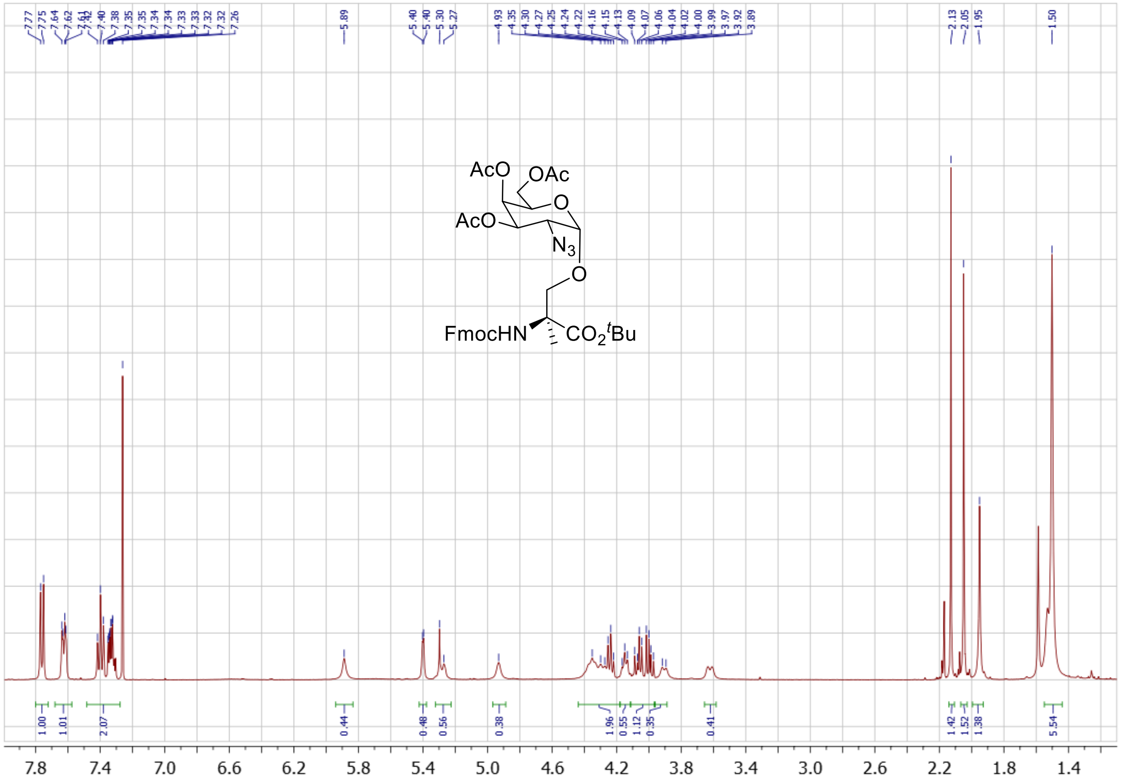

^{13}C NMR 100 MHz in $CDCl_3$ registered at 298K for **compound 11**

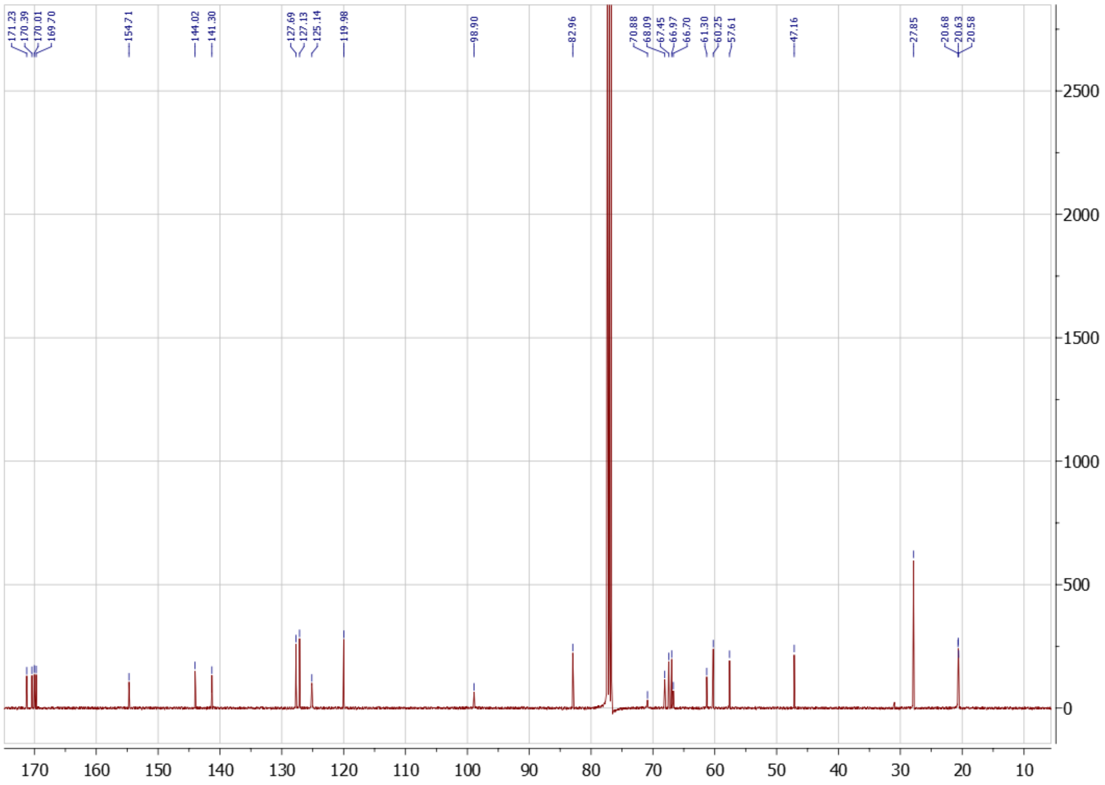

COSY in $CDCl_3$ registered at 298K for **compound 11**

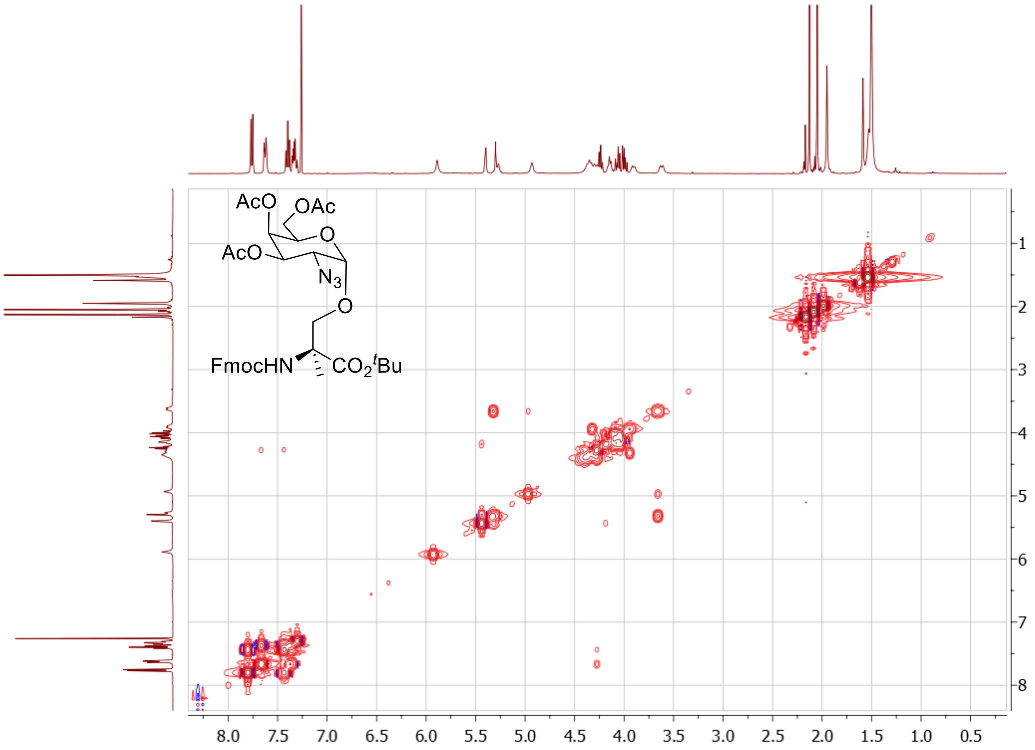

HSQC in $CDCl_3$ registered at 298K for **compound 11**

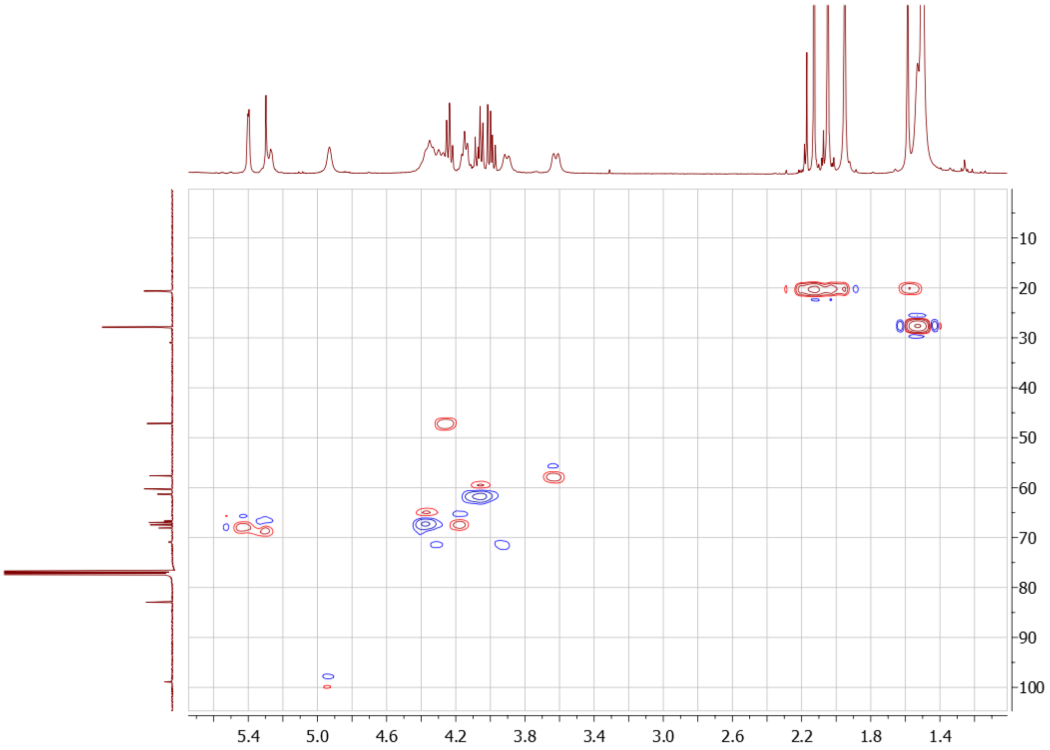

^{1}H NMR 400 MHz in $CDCl_3$ registered at 298K for **compound 12**

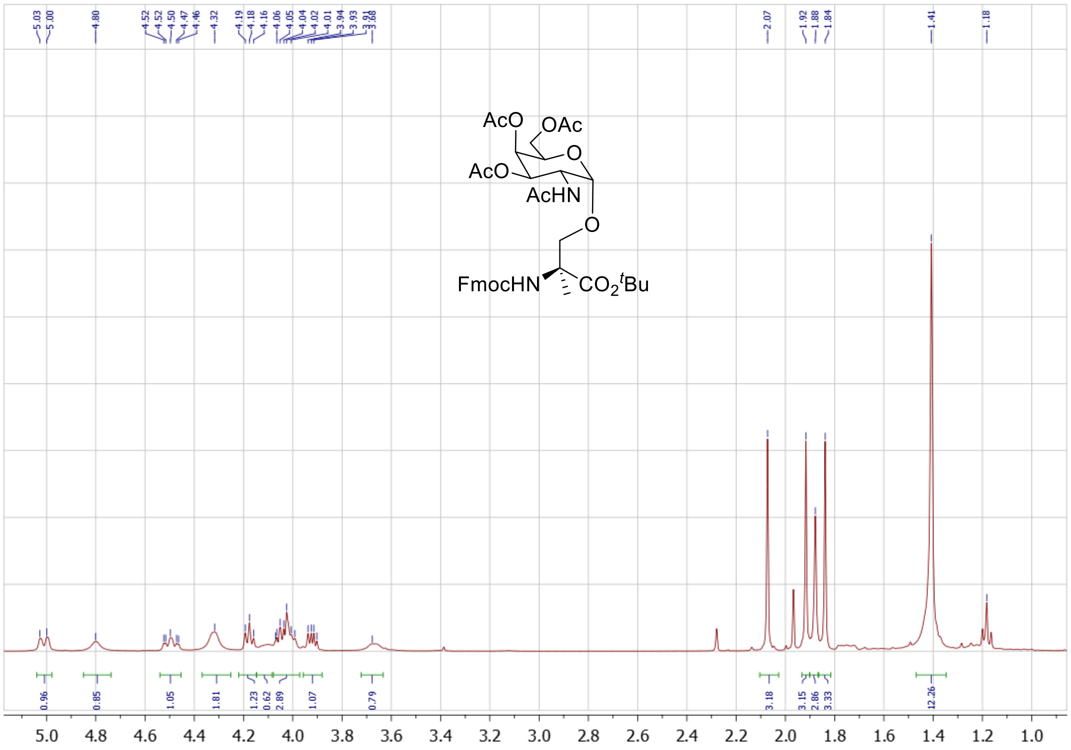

^{13}C NMR 100 MHz in $CDCl_3$ registered at 298K for **compound 12**

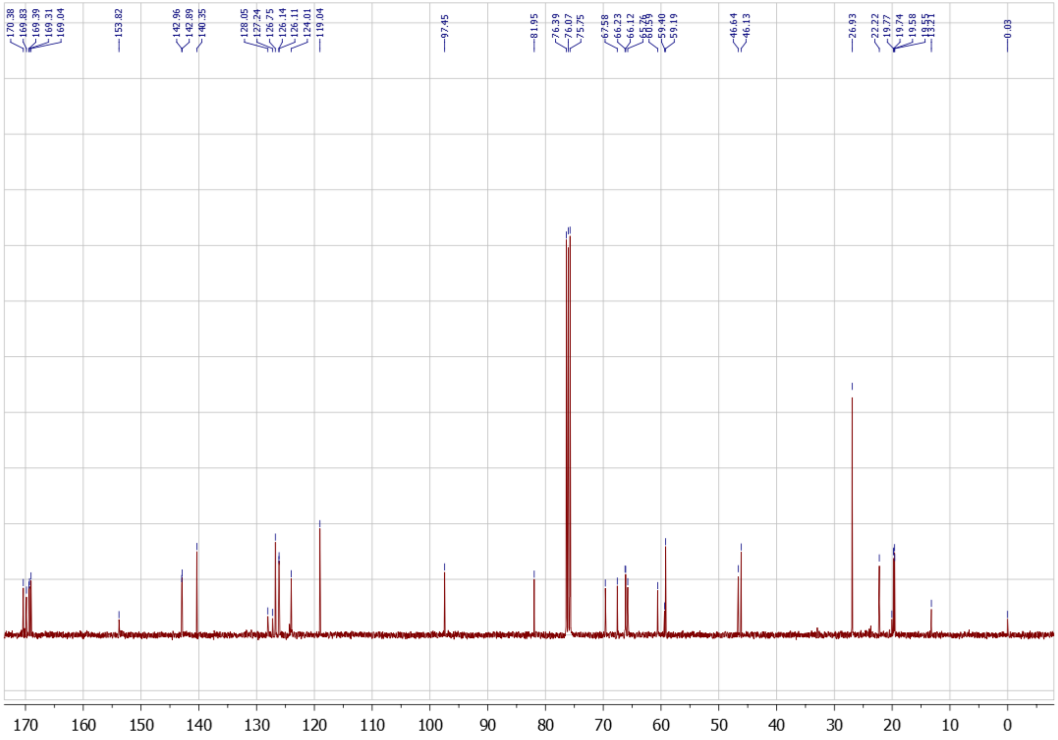

COSY in $CDCl_3$ registered at 298K for **compound 12**

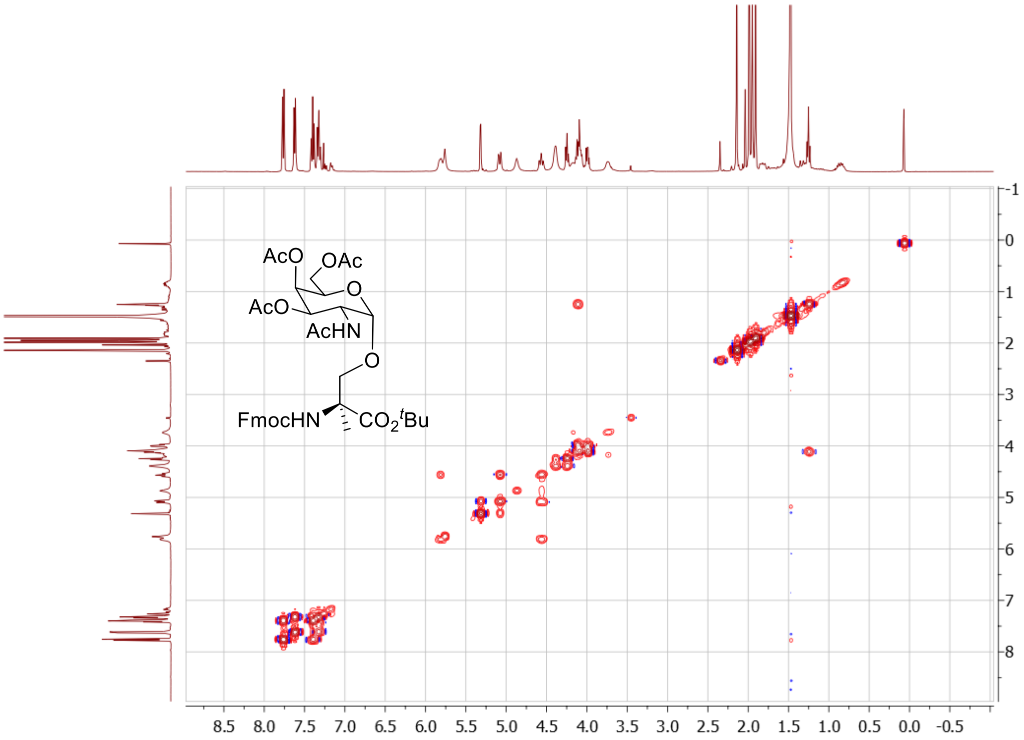

HSQC in $CDCl_3$ registered at 298K for **compound 12**

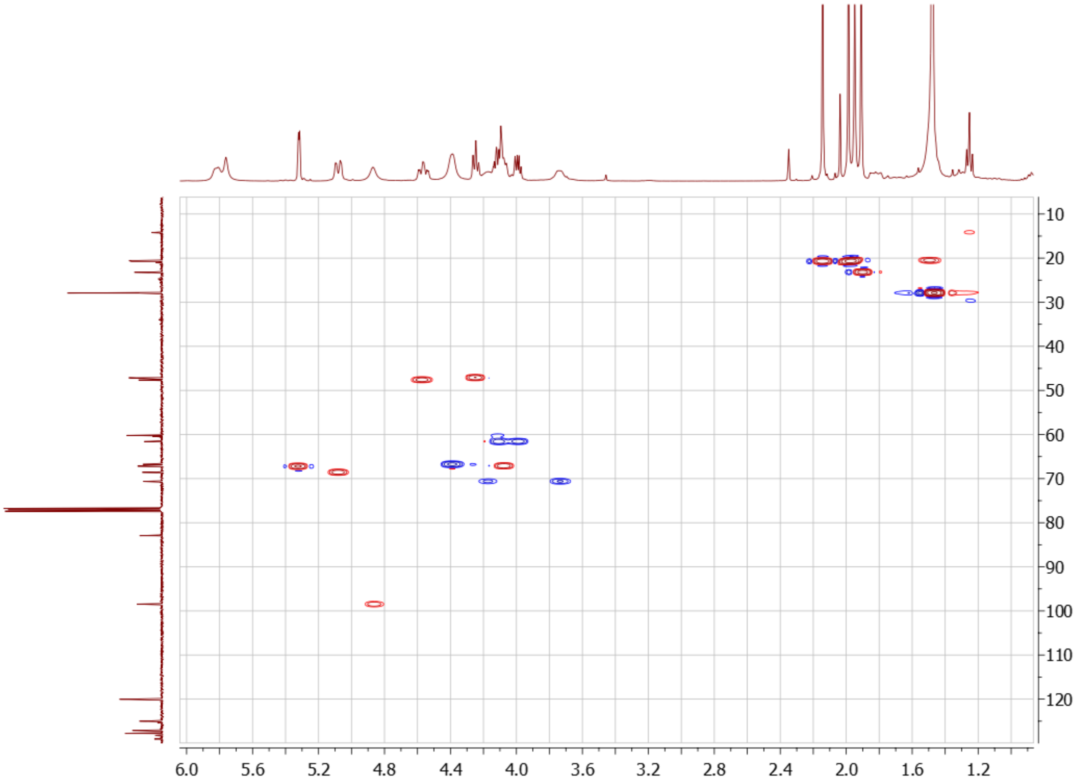

^{1}H NMR 400 MHz in $CDCl_3$ registered at 298K for **compound 13**

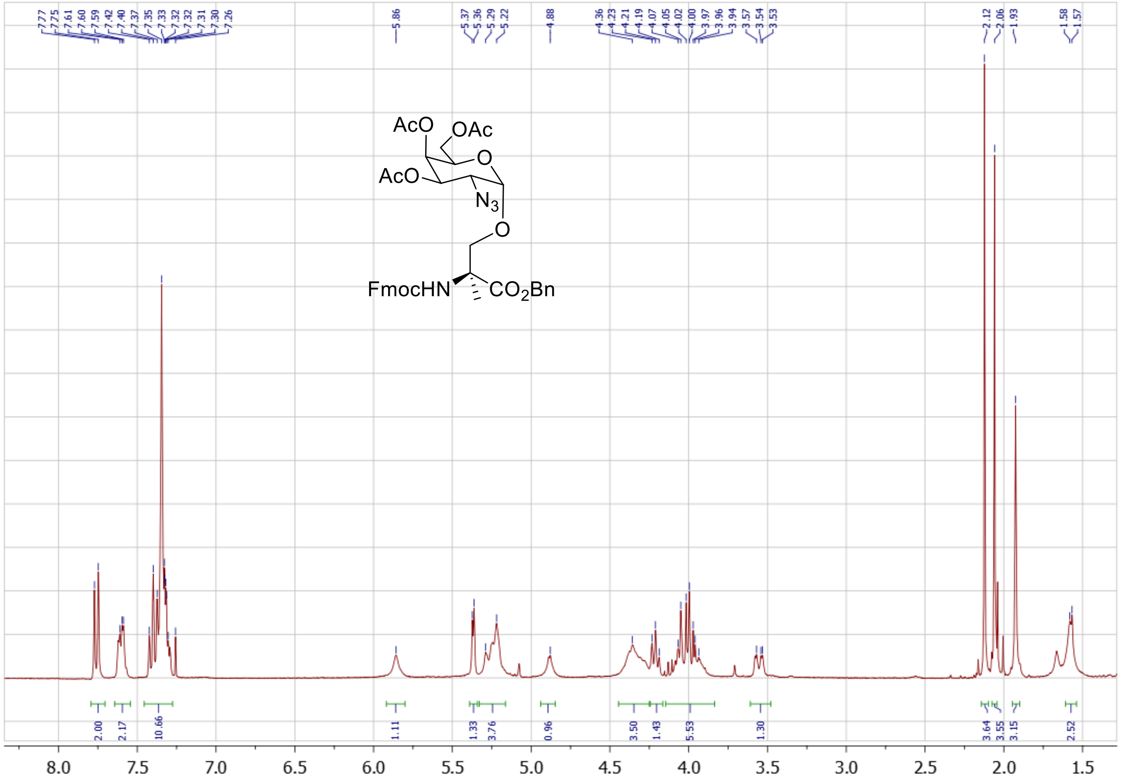

^{13}C NMR 100 MHz in $CDCl_3$ registered at 298K for **compound 13**

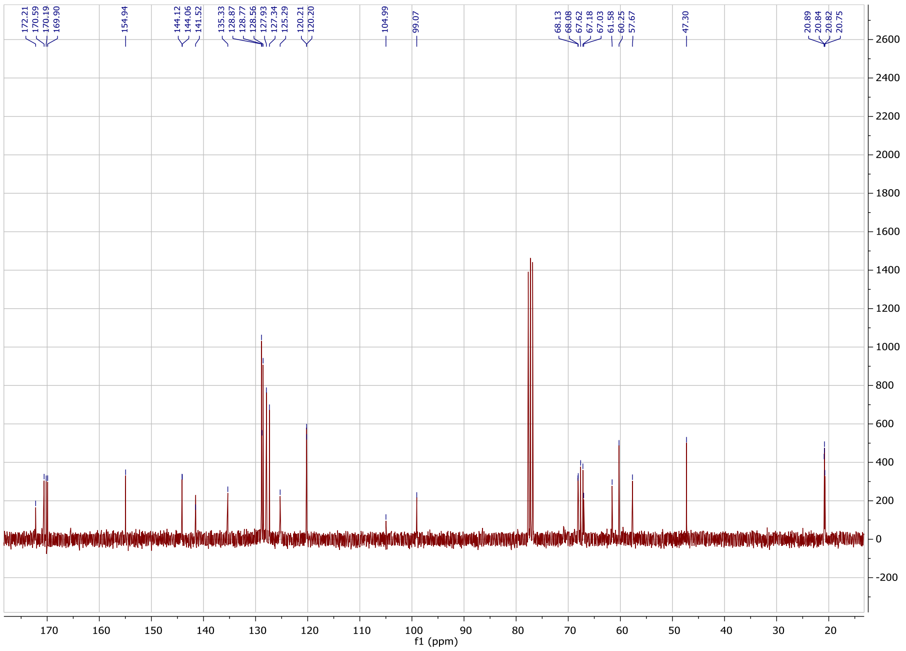

COSY in $CDCl_3$ registered at 298K for **compound 13**

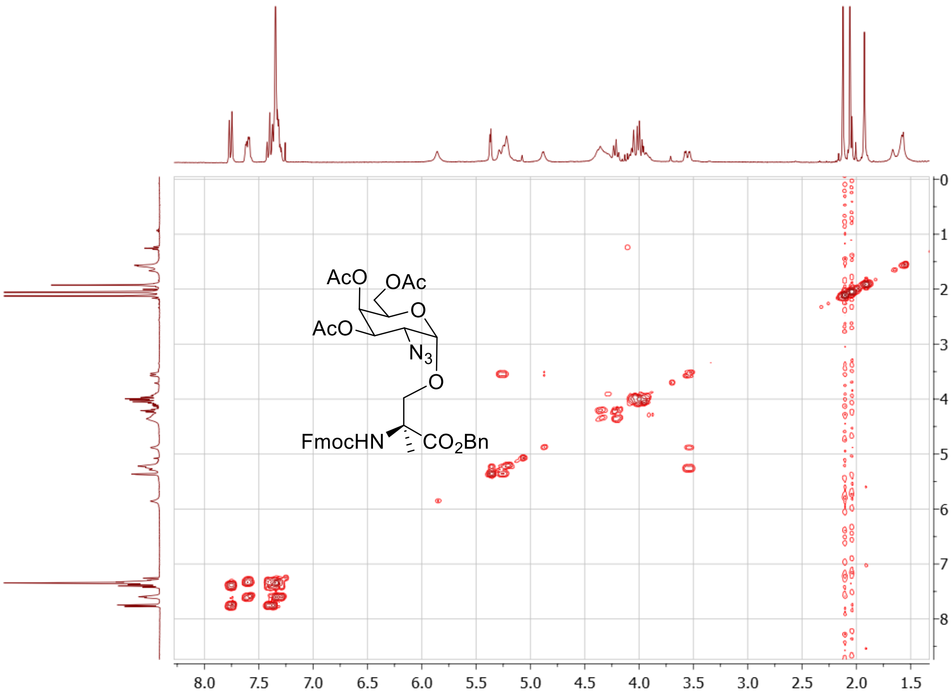

HSQC in $CDCl_3$ registered at 298K for **compound 13**

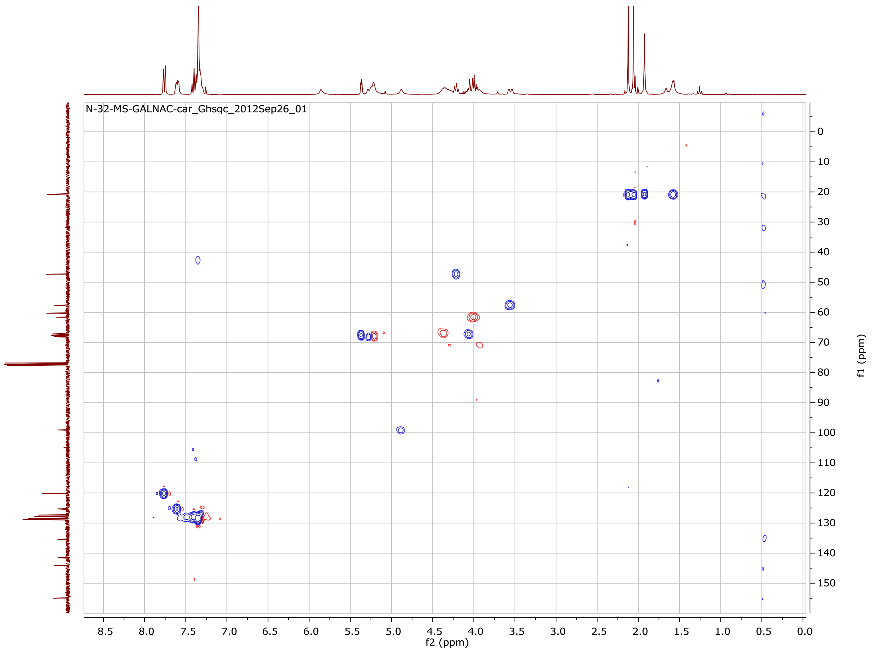

^{1}H NMR 400 MHz in $CDCl_3$ registered at 298K for **compound 14**

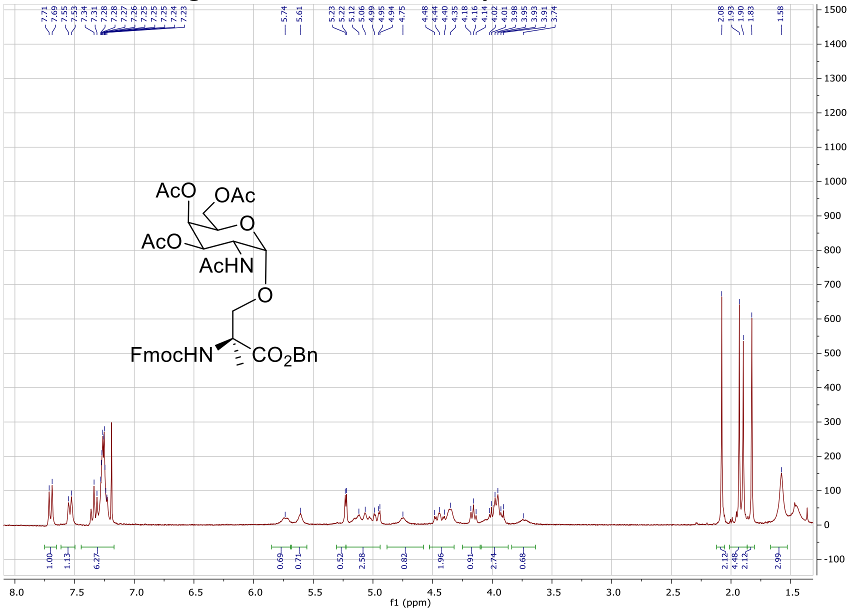

^{13}C NMR 100 MHz in $CDCl_3$ registered at 298K for **compound 14**

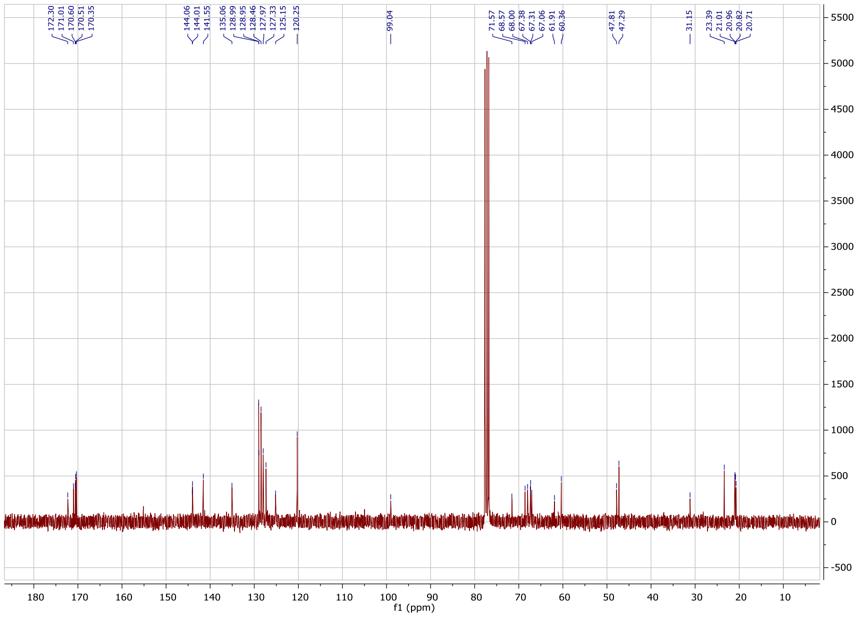

COSY in $CDCl_3$ registered at 298K for **compound 14**

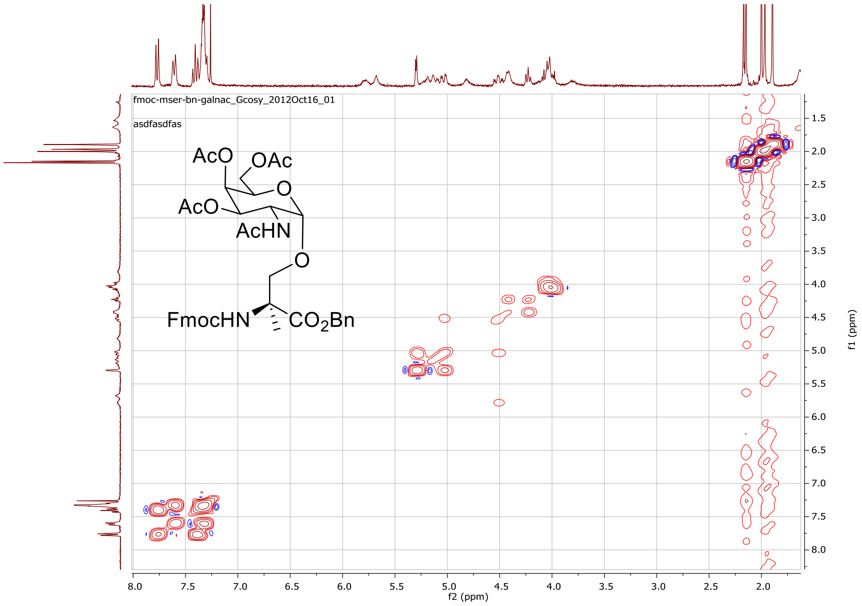

HSQC in $CDCl_3$ registered at 298K for **compound 14**

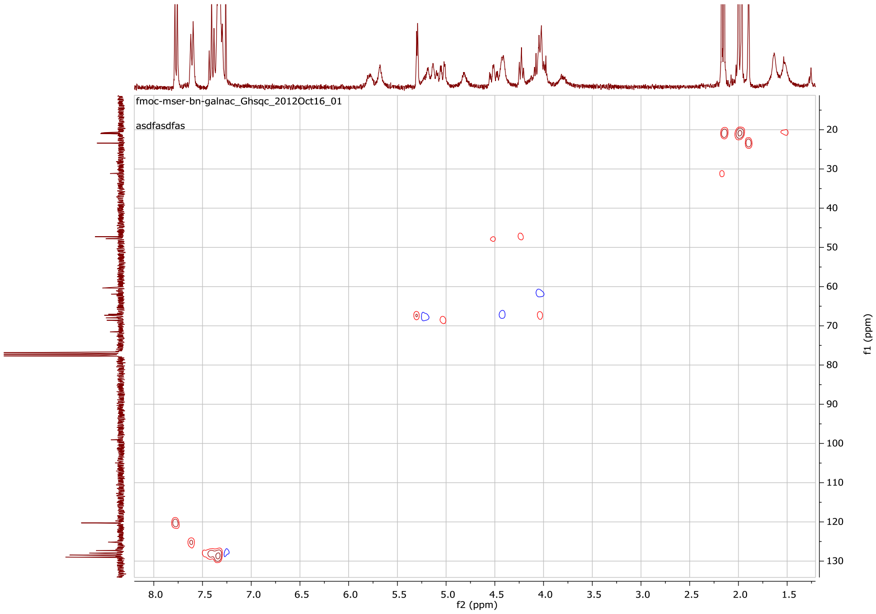

Table S1. Comparison of the experimental and MD simulation derived distances for glycopeptides **g1** and **g2**. Distances are given in Å.

	compound **g2**		compound **g1**	
	Exptl.	MD-tar	Exptl.	MD-tar
NH_{Arg}-$NH_{Thr/MeSer}$	≥3	3.1	≥3	3.1
NH_{Arg}-$H\alpha_{Arg}$	overlapping	2.9	2.7	2.9
NH_{Arg}-$H\alpha_{Thr}$	--		2.4	
NH_{Thr}-$H\alpha_{Thr}$	--		2.6	
$NH_{Thr/MeSer}$-$H\alpha_{Asp}$	2.7	2.5	2.2	2.5
NH_{Asp}-$H\alpha_{Asp}$	overlapping	2.9	2.7	2.9
NH_{Asp}-$H\alpha_{Pro}$	2.2	2.2	2.2	2.2
NH_{Asp}-$NH_{Thr/MeSer}$	≥3	3.0	≥3	3.0

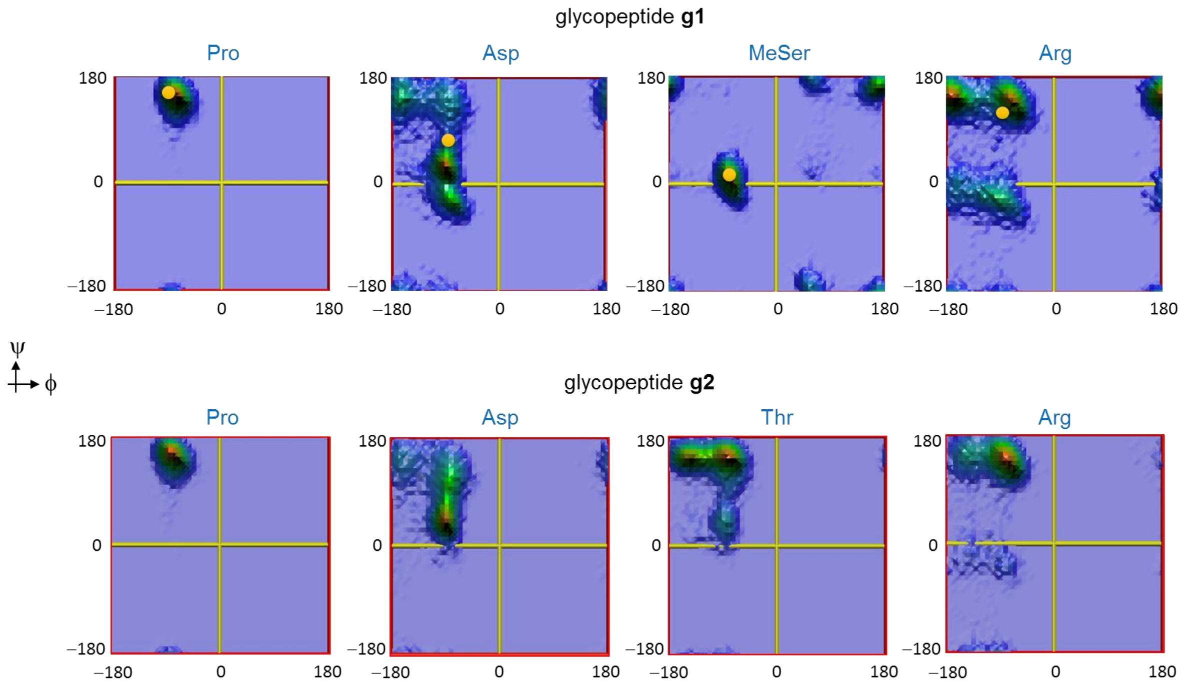

Figure S3. Distributions of the peptide backbone (ϕ/ψ) obtained through the 20 ns MD-tar simulations in explicit water for glycopeptides **g1** and **g2**. The yellow circles (upper panel) correspond to the conformation found in the crystal structure for glycopeptide APDT(αGalNAc)RP bound to scvF-SM3 (pdb entry: 5a2k).

Table 2. ELISA anti-MUC1 and anti-T_{helper} antibody titers[a] after 3rd and 4th immunizations with vaccine **1** (this work) the vaccine reported by Boons with threonine, **1-Thr** (Boons and co-workers *Proc. Natl. Acad. Sci. USA* **2012**, *109*, 261–266).

Immuni-zation[b]	IgG total MUC1 3rd imm	IgG total MUC1 endpoint	IgG1 MUC1 endpoint	IgG2a MUC1 endpoint	IgG2b MUC1 endpoint	IgG3 MUC1 endpoint	IgM MUC1 endpoint	IgG total T_{helper} endpoint
**PBS**	0	0	0	0	0	0	100	50
**EL**	500	1500	200	300	1000	3700	1500	100
1	18400	19000	4900	1900	9900	15000	9600	1000
**EL**	1800	1200	700	0	900	100	100	100
**1-Thr**	28500	59100	17800	7000	26800	11300	100	300

[a] Anti-MUC1 and anti-T_{helper} antibody titers are presented as median values for groups of mice. ELISA plates were coated with BSA-MI-CTSAPDT(α-D-GalNAc)RPAP conjugate for anti-MUC1 antibody titers or NeutrAvidin-biotin-T_{helper} for anti-T_{helper} antibody titers (see main text). Titers were determined by linear regression analysis, with plotting of dilution versus absorbance. Titers are defined as the highest dilution yielding an optical density of 0.1 or greater relative to normal control mouse sera. [b] Liposomal preparations were employed. EL stands for empty liposomes.

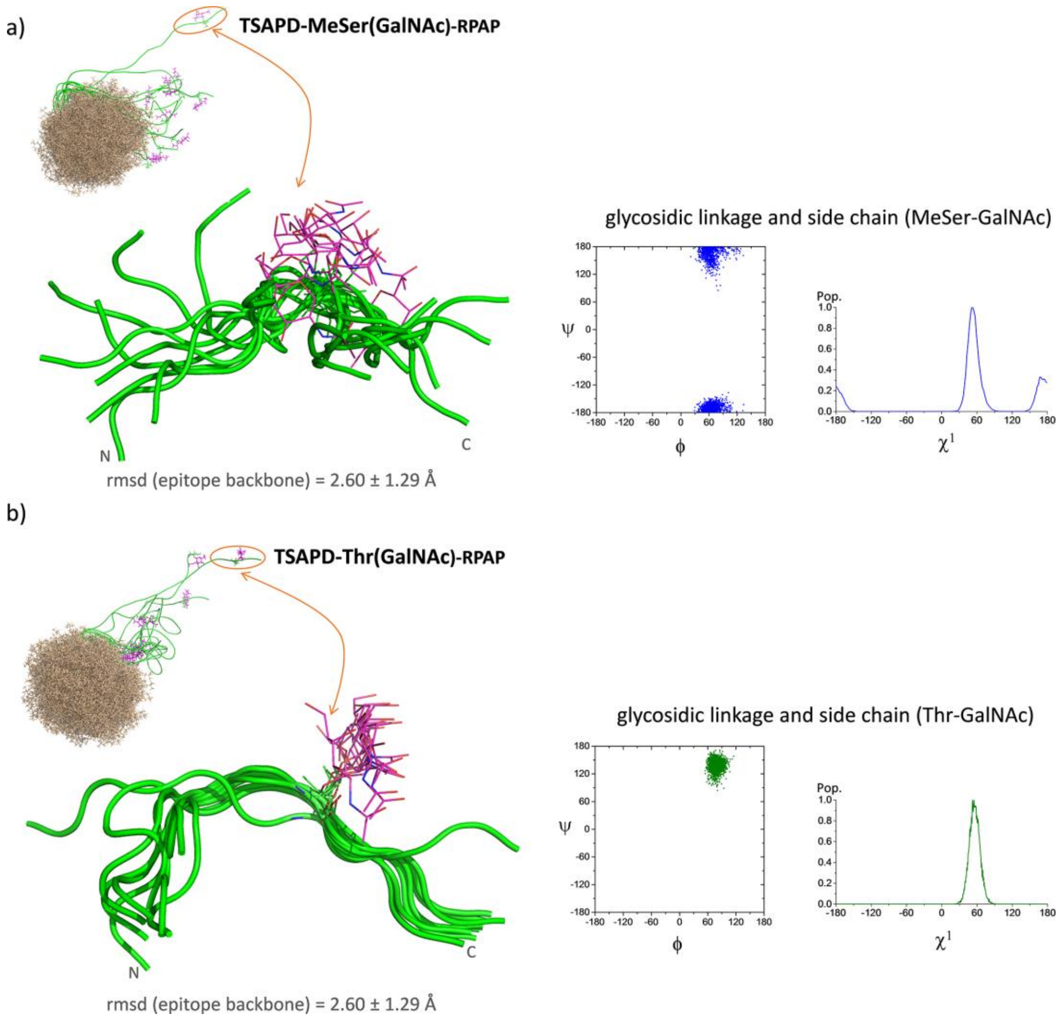

Figure S4. Ensembles obtained for lipoglycopeptide **1** (upper panel) and its analogue with threonine (bottom panel) through the 100 ns MD-tar simulations in water, together with the glycosidic linkage and side chain conformation of the glycosylated residue. The peptide backbone is shown in green and the carbons of the GalNAc residue in purple. Only the 10-mer MUC1 epitopes are shown for clarity. rmsd = root-mean-square deviation.

a)

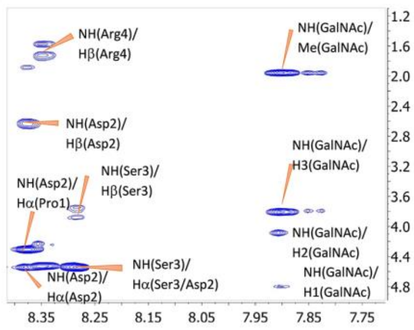

b)

distances	Exptl.	DM-tar
NHArg-NHSer	not observed	4.3
NHArg-HαArg	2.8	2.9
NHArg-HαSer	2.3	2.3
NHSer-HαSer	2.7	2.9
NHSer-HαAsp	2.2	2.6
NHAsp-HαAsp	overlapping	2.9
NHAsp-HαPro	overlapping	2.3
NHAsp-NHSer	overlapping	2.6

c)

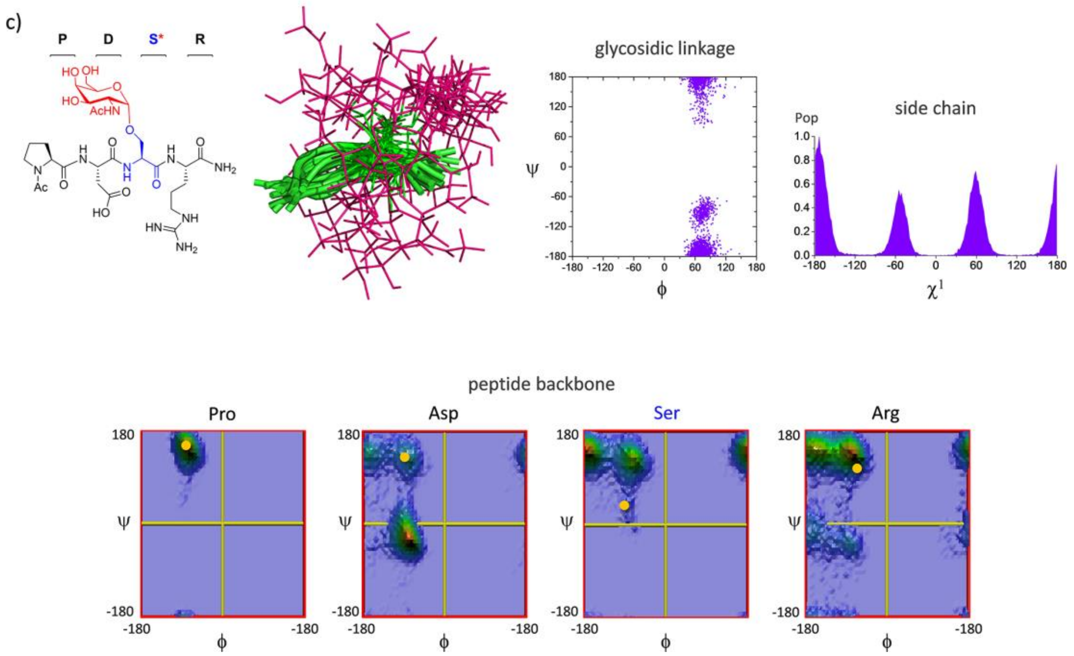

Figure S5. (a) Section of the 500 ms 2D NOESY spectrum (400 MHz) of glycopeptide **PDS(GalNAc)R** in H_2O/D_2O (9:1) at 25 °C and pH=5.6, showing the amide cross-peaks. The NOE contacts are represented as positive cross-peaks (blue color). (b) Comparison of the experimental and 20 ns MD-tar derived distances in explicit water for glycopeptide **PDS(GalNAc)R**. Distances are given in Å. (c) Ensembles obtained for glycopeptide **PDS(GalNAc)R** through the 20 ns MD-tar simulations in water, together with the conformation of the peptide backbone. The ϕ/ψ glycosidic linkage distribution and the distribution for the side chain of serine residue are also shown. The peptide backbone is shown in green and the carbons of the GalNAc residue in purple.

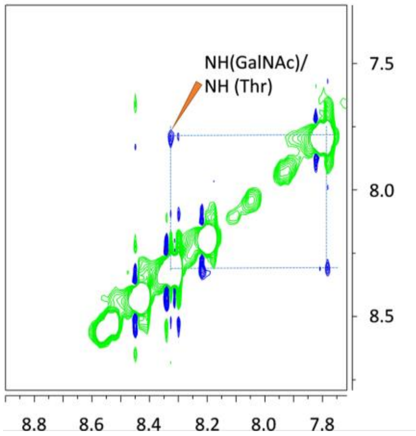

Figure S6. Section of the 500 ms 2D NOESY spectrum (400 MHz) of glycopeptide **g2** in H_2O/D_2O (9:1) at 25 °C and pH=5.6, showing the NOE cross-peak between the NH of GalNAc and the NH of threonine. This NOE is characteristic of the 'eclipsed' conformation of the glycosidic linkage.[S18] Diagonal peaks and exchange cross-peaks connecting NH protons and water are negative (green color). The NOE contacts are represented as positive cross-peaks (blue color).

REFERENCES

S1 C. Plattner, M. Höfener and N. Sewald, *Org. Lett.,* 2011, **13**, 545.

S2 M. Liu, V. G. Young Jr, S. Lohani, D. Live and G. Barany, *Carbohydr. Res.* 2005, **340**, 1273.

S3 C. Aydillo; G. Jiménez-Osés, J. H. Busto, J. M. Peregrina, M. M. Zurbano and A. Avenoza, *Chem. Eur. J.,* 2007, **13**, 4840.

S4 D. A. Case, T. A. Darden, T. E. Cheatham III, C. L. Simmerling, J. Wang, R. E. Duke, R. Luo, R. C. Walker, W. Zhang, K. M. Merz, B. Roberts, S. Hayik, A. Roitberg, G. Seabra, J. Swails, A. W. Götz, I. Kolossváry, K. F. Wong, F. Paesani, J. Vanicek, R. M. Wolf, J. Liu, X. Wu, S. R. Brozell, T. Steinbrecher, H. Gohlke, Q. Cai, X. Ye, J. Wang, M.-J. Hsieh, G. Cui, D. R. Roe, D. H. Mathews, M. G. Seetin, R. Salomon-Ferrer, C. Sagui, V. Babin, T. Luchko, S. Gusarov, A. Kovalenko and P. A. Kollman, AMBER 12; University of California: San Francisco, 2012.

S5 Wang, J., Wolf, R.M., Caldwell, J.W., Kollman, P.A. and Case, D.A., J. Comput. Chem., 2004, **25**, 1157.

S6 C. I. Bayly, P. Cieplak, W. Cornell and P. A. Kollman, *J. Phys. Chem.*, 1993, **97**, 10269.

S7 Gaussian 09, Revision A.1, M. J. Frisch, G. W. Trucks, H. B. Schlegel, G. E. Scuseria, M. A. Robb, J. R. Cheeseman, G. Scalmani, V. Barone, B. Mennucci, G. A. Petersson, H. Nakatsuji, M. Caricato, X. Li, H. P. Hratchian, A. F. Izmaylov, J. Bloino, G. Zheng, J. L. Sonnenberg, M. Hada, M. Ehara, K. Toyota, R. Fukuda, J. Hasegawa, M. Ishida, T. Nakajima, Y. Honda, O. Kitao, H. Nakai, T. Vreven, J. A. Montgomery, Jr., J. E. Peralta, F. Ogliaro, M. Bearpark, J. J. Heyd, E. Brothers, K. N. Kudin, V. N. Staroverov, R. Kobayashi, J. Normand, K. Raghavachari, A. Rendell, J. C. Burant, S. S. Iyengar, J. Tomasi, M. Cossi, N. Rega, J. M. Millam, M. Klene, J. E. Knox, J. B. Cross, V. Bakken, C. Adamo, J. Jaramillo, R. Gomperts, R. E. Stratmann, O. Yazyev, A. J. Austin, R. Cammi, C. Pomelli, J. W. Ochterski, R. L. Martin, K. Morokuma, V. G. Zakrzewski, G. A. Voth, P. Salvador, J. J. Dannenberg, S. Dapprich, A. D. Daniels, O. Farkas, J. B. Foresman, J. V. Ortiz, J. Cioslowski, and D. J. Fox, Gaussian, Inc., Wallingford CT, 2009.

S8 D. P. Tieleman, D. van der Spoel and H.J.C. Berendsen, *J. Phys. Chem. B*, 2000, **104**, 6380.

S9 C. J. Dickson, B. D. Madej, A. A. Skjevik, R.M. Betz, K. Teigen, I. R. Gould and R. C. Walker, *J. Chem. Theory Comput.*, 2014, **10**, 865.

S10 J. A. Maier, C. Martinez, K. Kasavajhala, L. Wickstrom, K. E. Hauser and C. Simmerling, *J. Chem. Theory Comput.*, 2015, **11**, 3696.

S11 K. N. Kirschner, A. B. Yongye, S. M. Tschampel, J. González-Outeiriño, C. R. Daniels, B. L. Foley and R. J. Woods, *J. Comput. Chem.*, 2008, **29**, 622.

S12 W. L. Jorgensen, J. Chandrasekhar, J. D. Madura, R. W. Impey and M. L. Klein, *J. Chem. Phys.,* 1983, **79**, 926.

S13 T. A. Andrea, W. C. Swope, H. C. Andersen, *J. Chem. Phys.,* 1983, **79**, 4576.

S14 Darden, T., York, D. and Pedersen, L., *J. Chem. Phys.,* 1993, **98**, 10089.

S15 D. A. Pearlman and P. A. Kollman, *J. Mol. Biol.*, 1991, **220**, 457.

S16 K. B. Horwitz, M. E. Costlow and W. L. McGuire, *Steroids,* 1975, **26***,* 785.

S17 P. Mukherjee, A. R. Ginardi, T. L. Tinder, C. J. Sterner and S. J. Gendler, *Clin. Cancer Res.,* 2001, **7**, 848s.

S18 F. Corzana, J. H.Busto, G. Jiménez-Osés, M. García de Luis, J. L. Asensio, J. Jiménez-Barbero, J. M. Peregrina and A. Avenoza, *J. Am. Chem. Soc.*, 2007, **129**, 9458.
